# Approaching Hypothetical RbTl in Experiments and Theory – X-ray Structure Determination of Cs_1–*x*_Rb*_x_*Tl (*x* = 0.18, 0.42) and a Solid Solution K_1–*x*_Rb*_x_*Tl (*x* ≤ 0.69)

**DOI:** 10.1021/acs.inorgchem.4c05305

**Published:** 2025-04-03

**Authors:** Vanessa F. Schwinghammer, Saleem A. Khan, Susanne M. Tiefenthaler, Tomáš Kovářík, Ján Minár, Stefanie Gärtner

**Affiliations:** † Institute of Inorganic Chemistry, University of Regensburg, Universitätsstraße 31, Regensburg 93053, Germany; ‡ Central Analytics, University of Regensburg, Universitätsstaße 31, Regensburg 93053, Germany; § New Technologies Research Center, University of West Bohemia, Pilsen 301000, Czech-Republic

## Abstract

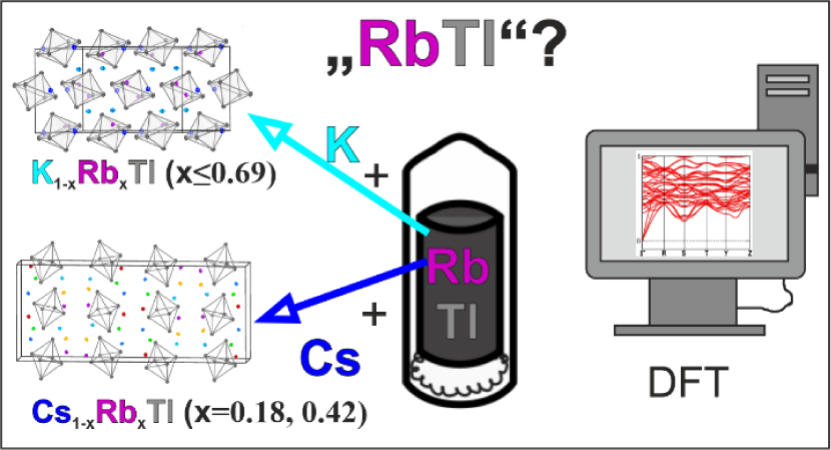

Although the binary alkali metal thallides ATl with A = Li, Na, K, and Cs have been reported in the literature, binary RbTl at ambient pressure is still missing. Experiments with a 1:1 ratio of Rb:Tl, either according to Zintl’s procedure in low-temperature experiments in liquid ammonia or classical solid-state synthesis at high temperature, did not result in the desired product. Therefore, several ternary compositions with mixtures of K/Rb and Cs/Rb have been prepared. For K/Rb mixtures, a solid solution in the KTl structure type, up to a proportion of 69% rubidium, could be obtained. Site occupancy preferences for rubidium on the alkali metal sites in the KTl type are observed in experiments and supported by theoretical calculations. In contrast to Rb/K mixtures being realizable in the KTl structure type, Rb/Cs mixtures did not allow for the isolation of materials according to the CsTl structure type. Instead, two new monoclinic compounds could be isolated (Cs_0.82_Rb_0.18_Tl: *C*2/*c*, *a* = 14.4136(4) Å, *b* = 11.1678(3) Å, *c* = 40.8013(11) Å, β = 96.353(2)°, *V* = 6527.4(3) Å^3^; Cs_0.58_Rb_0.42_Tl: *C*2/*c*, *a* = 14.2610(3) Å, *b* = 11.1116(2) Å, *c* = 27.5589(7) Å, β = 104.056(2)°, *V* = 4236.30(17) Å^3^). Detailed DFT calculations on both binary and mixed cation systems were performed and support the experimental results.

## Abstract 2

Binary RbTl is not accessible in experiments, neither by Zintl’s route in liquid ammonia nor from the reaction of the elements. The approximation of this missing compound is discussed, starting from the existing binary compounds KTl and CsTl. While the K/Rb mixture resulted in a solid solution up to 69% in the KTl structure type, the Cs/Rb mixture yielded two novel monoclinic compounds, Cs_1−*x*_Rb_*x*_Tl (*x* = 0.18, 0.42). Additional DFT calculations for the stability of those mixtures, including the still hypothetical RbTl, were carried out.

## 1 Introduction

In 1932, E. Zintl and W. Dullenkopf reported on the binary phase NaTl as the first example of a compound with formally negatively charged thallium, the so-called thallides.^1,2^ The formal electron transfer from the less electronegative metal sodium to the more electronegative thallium results in a diamond-like thallium substructure, which was interpreted in terms of the pseudoelement approach introduced by W. Klemm.^1,3^ While located to the left of the so-called Zintl border between group 13 and group 14, introduced by F. Laves, textbook known NaTl is still referred to as the first Zintl phase and therefore set a milestone for the chemistry of Zintl compounds in general.^4^ NaTl can be prepared by using classical high-temperature solid-state synthesis. At the same time, a stoichiometry range is given in the related binary phase diagram.^5,6^ In addition, E. Zintl himself also prepared NaTl in a low-temperature solution route by reducing thallium(I) iodide with sodium in liquid ammonia.^1,7,8^ While the heavier congeners of the alkali metals, potassium and cesium, can be prepared by high-temperature synthesis, the low-temperature route has not yet been reported for these heavier alkali metals. Interestingly, KTl and CsTl do not yield NaTl-analogue compounds; instead [Tl_6_]^6–^ octahedra are present in the unit cells of the crystal structures of the latter compounds.^9,10^ Applying Wade’s rules for these clusters, a lack of electrons can be identified compared to an octahedral *closo* cluster, which would afford an 8-fold negative charge for the [Tl_6_] entity.^11−13^ In KTl and CsTl, (2*n*) skeletal electrons of [Tl_6_]^6–^ classify them as hypoelectronic.^14^ This affects the shape of the clusters, as it significantly deviates from an ideal octahedral shape. The observed compression was explained as a result of a Jahn–Teller distortion.^9,10^ The binaries KTl and CsTl crystallize in different orthorhombic space groups (KTl: *Cmce*; CsTl: *Fddd*), but both still include [Tl_6_]^6–^ clusters as anionic moieties. Recently, it was shown that the combination of potassium and cesium in ternary Cs_1–*x*_K_*x*_Tl approaches allows for the formation of pentagonal bipyramidal-shaped [Tl_7_]^7–^ clusters in Cs_3.45_K_3.55_Tl_7_ and Cs_7.29_K_5.71_Tl_13_.^15^ In general, mixing alkali metals increases the variety of thallide compounds, which are not yet accessible in binary materials.^16^

Concerning the thallides in an alkali metal-thallium ratio of 1:1, the absence of RbTl under ambient conditions is remarkable, especially as the remaining binaries LiTl, NaTl, KTl, and CsTl are long-time known.^1,7−10,17^ For RbTl, only a high-pressure phase with the NaTl structure has been mentioned.^18,19^ The binary phase diagram of the Rb–Tl system contains RbTl_3_ (=Rb_4_Tl_13_), RbTl_2_ (=Rb_15_Tl_27_), and Rb_4_Tl_6_ (=Rb_8_Tl_11_) but does not include an equimolar compound.^5,20−22^ In addition, a further binary compound Rb_49_Tl_109.67_ was later reported, which also does not appear in the phase diagram.^23^ The phase diagram for the binary system Cs–Tl again does not show an equimolar compound, but Dong and Corbett reported on CsTl already in 1996.^10,24^ These examples nicely demonstrate that the absence of a binary compound in a phase diagram does not contradict its existence.

In this perspective, we report on attempts to prepare binary RbTl by high-temperature synthesis as well as low-temperature approaches in liquid ammonia. Additionally, RbTl is approximated in ternary approaches involving mixed alkali metals A_1–*x*_Rb_*x*_Tl (A = K or Cs). The homogeneity range of K_1–*x*_Rb_*x*_Tl (*x* ≤ 0.69) is discussed, and two new monoclinic compounds, Cs_1–*x*_Rb_*x*_Tl (*x* = 0.18, 0.42) are presented. In addition, DFT calculations on the binaries and ternaries have been performed to support the experimental findings.

## 2 Experimental Section

### 2.1 Preparation

Potassium (Sigma-Aldrich, purity 98%, under mineral oil) was segregated for purification. Rubidium and cesium were obtained by the reduction of RbCl or CsCl with elemental calcium and afterward purified through distillation twice.^25^ Thallium drops (ABCR, purity of 99.999%) were used without further purification and stored under an inert gas atmosphere.

#### 2.1.1 High-Temperature Syntheses

The high-temperature solid-state syntheses were carried out in sealed tantalum ampoules using the elements under an argon atmosphere. The sealed ampoules were placed in quartz glass tubes (QSIL GmbH, Ilmenau, Germany) and sealed under an argon atmosphere. Different temperature programs were used after holding at 773.15 K (or 673.15 K) for 48 h.

Cooling 1: cooling to room temperature at a 5 K per hour cooling rate.

Cooling 2a: quenching the ampoule in water.

Cooling 2b: quenching the ampoule in water, annealing at 393.15 K for 2 weeks, subsequently at 373.15 K for 1 week, and then cooling to room temperature at a 5 K per hour cooling rate.

Cooling 3: quenching in liquid nitrogen.

The received products are sensitive to moisture and oxygen. Therefore, they were stored in a glovebox (Labmaster 130 G, Fa. M. Braun, Garching, Germany).

Compositions of prepared samples and the temperature programs used: KTl (Cooling1), CsTl (Cooling1), K_3_Rb_7_Tl_7_ (Cooling2b), K_5_Rb_5_Tl_7_ (Cooling2b), K_5_RbTl_6_ (Cooling1), K_2_RbTl_3_ (Cooling1), Cs_2_RbTl_3_ (Cooling2a), Cs_6_Rb_4_Tl_10_ (Cooling2a), Cs_7_Rb_3_Tl_7_ (Cooling2b), RbTl (Cooling3), and Rb_1.1_Tl (Cooling3). The complete sample/temperature program/result listing is given in Table S3.

#### 2.1.2 Low-Temperature Syntheses

Liquid ammonia was condensed on sodium and kept under argon, cooled by a dry ice/ethanol bath. Anhydrous ammonia (5 mL) was condensed onto the mixture of reactants ((1) 2Rb + TlBF_4_, (2) 2Rb + TlPF_6_, and (3) 2Rb + TlBr)) in reaction vessels that had been baked-out three times and stored at 233.15 K. The approaches were prepared and stored at 233.15 K. After 2–5 days, ammonia was evaporated, and the residue was characterized by X-ray powder diffraction (see Supporting Information Chapter 4).

### 2.2 Single-Crystal X-Ray Diffraction

A small number of crystals were transferred into dried mineral oil. From these, a suitable crystal was selected and mounted on a Rigaku SuperNova diffractometer (X-ray: Mo/Ag microfocus, AtlasS2 detector) or a Rigaku XtaLAB Synergy R DW system diffractometer (X-ray: Cu/Mo rotating anode, HyPix-Arc 150 detector) (Rigaku Polska Sp. z o. o. UI, Wroclaw, Poland) using MiTeGen loops. All data were collected at 123 K.

*CrysAlisPro* (ver. 171.43.105a) was used for data collection and data reduction.^26^ For the structure solution, *ShelXT* was used, and the subsequent data refinement was carried out with *ShelXL or olex2refine*.^27−30^*Olex*^*2*^ was used for visualization purposes, and the software *Diamond4* was chosen for the representation of the crystal structure.^31,32^ All atoms are depicted as ellipsoids with a 50% probability level.

Crystallographic data for the compounds have been deposited in the Cambridge Crystallographic Data Center CCDC, 12 Union Road, Cambridge CB2 1EZ, UK. Copies of the data can be obtained free of charge under the depository numbers CCDC 2295143 (KTl), 2295126 (K_0.86_Rb_0.14_Tl), (K_0.72_Rb_0.28_Tl), 2295115 (K_0.54_Rb_0.46_Tl), 2348700 (K_0.31_Rb_0.69_Tl), 2295021 (CsTl), 2295125 (Cs_0.58_Rb_0.42_Tl), and 2386360 (Cs_0.82_Rb_0.18_Tl).

### 2.3 Powder X-Ray Diffraction (PXRD)

Due to their sensitivity to air and moisture, all samples were prepared in sealed capillaries (Ø0.3 mm, WJM-Glas-Müller GmbH, Berlin, Germany). The data collection was carried out on an STOE Stadi P diffractometer (STOE, Darmstadt, Germany) (monochromatic Mo Kα1 radiation, λ = 0.70926 Å) equipped with a Dectris Mythen 1K detector. For visualization and indexation, the software *WinXPOW* and *Jana2006* were used.^33,34^

### 2.4 DSC Measurements

Differential scanning calorimetry (DSC) measurements were performed using a TA Instruments Q200 analyzer. DSC analysis was carried out under a flow of nitrogen (sample purge flow: N_2_ 40 mL/min). The sample was sealed in a fume hood using a TA Instruments Tzero hermetic aluminum pan, and four heating/cooling cycles were performed from 293.15 to 593.15 K at a 10 K/min rate.

The powder sample was homogenized by precalcination at 593 K in DSC mode to minimize baseline drift and reduce thermal artifacts.

### 2.5 DFT Calculations

To explore the theoretical aspects of Cs_1–*x*_Rb*_x_*Tl and K_1–*x*_Rb*_x_*Tl we used different methods, namely the projector-augmented wave method (PAW),^35^ implemented in the Vienna Ab initio simulation package (VASP)^36,37^ and the multiple scattering Korringa–Kohn–Rostoker (KKR) Green function method as implemented in the SPRKKR code.^38,39^ To observe the effect of disorder, the coherent potential approximation (CPA)^40^ implemented in the SPRKKR code was used. In both codes, our calculations are based on the Perdew, Burke, and Ernzerhof generalized gradient approximation (PBE-GGA).^41^ The calculations were performed in several successive steps. The geometry optimization and electronic structure calculations were conducted using the VASP code. All of the convergence parameters in the code were checked carefully.

In the VASP code, the geometries were relaxed using the conjugate gradient method, with forces estimated using the Hellmann–Feynman theorem. For structure relaxation, an energy cutoff of 700 eV and ISIF = 3 were adopted. The self-consistencies of the ground-state energies of K_1–*x*_Rb_*x*_Tl and Cs_1–*x*_Rb_*x*_Tl were obtained with an energy cutoff of 320 eV. For *k*-point sampling, an automatic *k*-mesh was used for both compounds, with 16 *k*-points in the irreducible Brillouin zone (IBZ), distributed according to a (3 × 3 × 6) and a (6 × 3 × 2) Monkhorst–Pack grid.^42^ For further density of state calculations of K_1–*x*_Rb_*x*_Tl, dense *k*-mesh was used by increasing the *k*-point grid to (4 × 4 × 7).

The energy and force convergence criteria were set at 10^–6^ eV and 10^–3^ eV/Å, respectively.

Additionally, the phonon frequencies of the hypothetical RbTl were calculated using first-principles phonon calculations with a finite displacement method^43,44^ implemented in phonopy code^45^ interfaced with the VASP package. The accuracy of the phonon calculation is sensitive to various technical parameters, including supercell size, force convergence symmetry, energy convergence, and atomic displacement. In the present calculation, we used default values of 1.0 for force convergence symmetry, an energy convergence criterion of 10^–6^ eV, and a default atomic displacement of 0.01 Å. Details on supercell size can be found in the Supporting Information (Table S15). In the phonon calculations, only the Gamma-point (Γ) was used for *k*-space sampling.

## 3 Results

### 3.1 Binary RbTl Approaches

First attempts at the synthesis of binary RbTl were carried out by the historical low-temperature experiments in anhydrous liquid ammonia. In these experiments, elemental rubidium and thallium(I) salts were reacted in a 2:1 ratio (Rb:Tl(I)X (X = Br, BF_4_, PF_6_)) at low temperature, which is a well-known preparation route for NaTl.^1^ The variation of the halides Tl(I)X (X = Cl, Br, I) to weakly coordinating anions such as [BF_4_]^−^ or [PF_6_]^−^ was also tested. All approaches resulted in the formation of elemental thallium and a rubidium salt, formed by Br^–^ or the weakly coordinating anion (see PXRD in Supporting Information Chapter 4). The low-temperature route, therefore, seems to be limited to NaTl.

**Figure 1:**
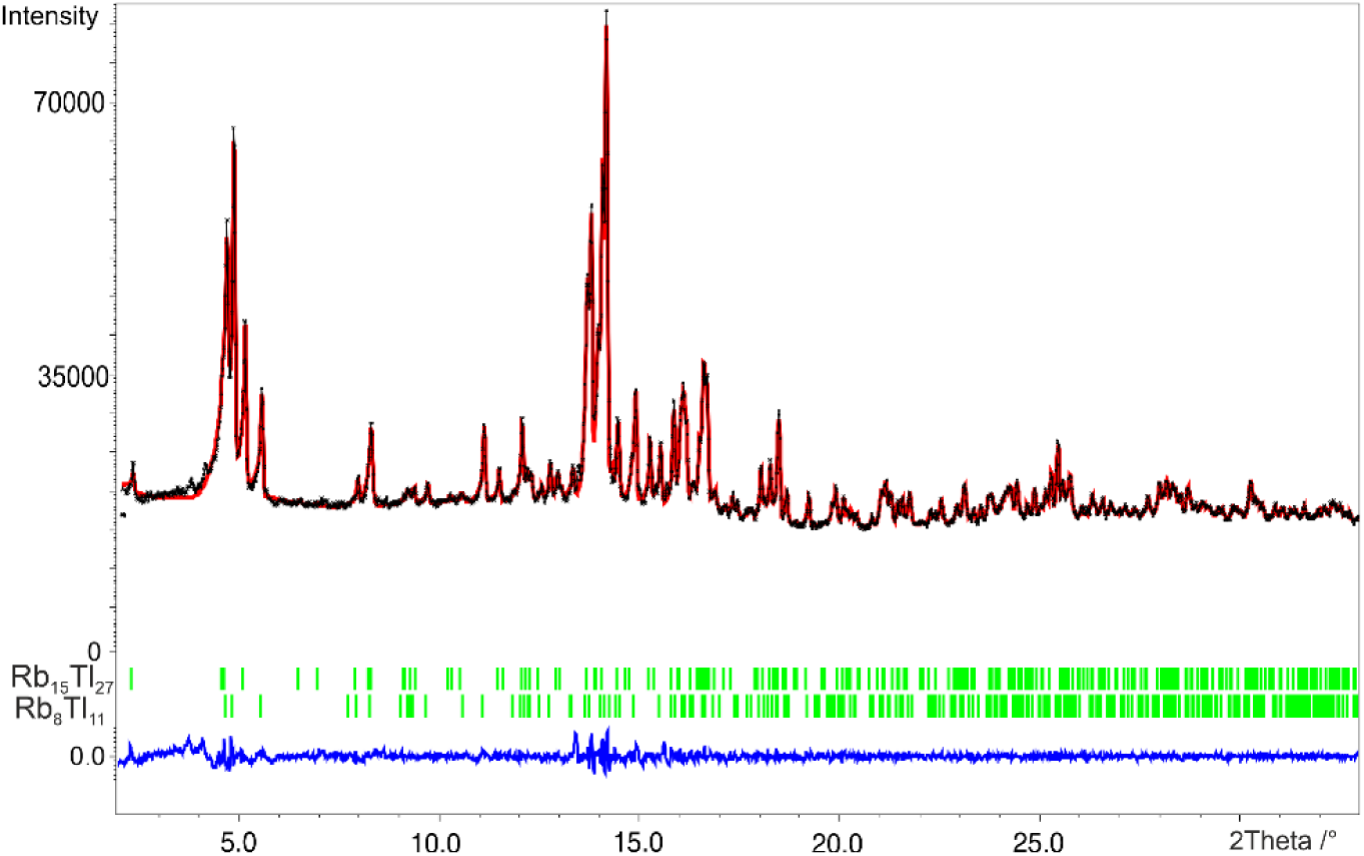
Measured powder diffraction pattern of the product of sample RbTl (black). The refinement was carried out with the LeBail algorithm. The calculated reflection positions are shown by the vertical bars (green) underneath the powder pattern. The curve at the bottom (blue) represents the difference plot. GOF = 1.76, *R*_p_ = 0.65, *R*_wp_ = 0.96. *x*-axis: 2Theta in °, *y*-axis: intensity.

As an alternative route, high-temperature solid-state reactions from the elements in different alkali metal-to-thallium ratios and subsequent quenching to 77 K in liquid nitrogen always yielded a mixture of Rb_8_Tl_11_ and Rb_15_Tl_27_ (Figure 1). Depending on the excess of alkali metal, elemental rubidium was also involved. These results indicate that binary RbTl is not accessible in experimental settings.

### 3.2 Partial Substitution of Potassium by Rubidium

Due to the fact that attempts to prepare binary RbTl have not succeeded so far, we tried to approximate this compound by ternary approaches with potassium or cesium. Combinations of rubidium and potassium yielded in a homogeneity range of the KTl structure type, up to a rubidium proportion of 69% (see crystallographic data in Table 1). As a side phase, K_8–*x*_Rb*_x_*Tl_11_ was always present, which was also reported for binary KTl.^9^

**Table 1:** Shortened Crystallographic Data of the Redetermined KTl at 123 K and Ternary Solid Solutions K_1–*x*_Rb*_x_*Tl (*x* ≤ 0.69)^a^

Empirical Formula	KTl^b^	K_0.86_Rb_0.14_Tl	K_0.72_Rb_0.28_Tl	K_0.54_Rb_0.46_Tl	K_0.31_Rb_0.69_Tl
CSD number	2295143	2295126	2295113	2295115	2348700
formula weight	243.482	249.87	256.36	264.66	275.25
temperature (K)	123				
crystal system	orthorhombic				
space group	*Cmce*				
*a* (Å)	15.2382(4)	15.3150(3)	15.3508(4)	15.4681(11)	15.5750(6)
*b* (Å)	14.9476(4)	15.0130(3)	15.0226(5)	15.1269(9)	15.2312(5)
*c* (Å)	8.0763(2)	8.1263(2)	8.1483(3)	8.2196(5)	8.2726(3)
volume (Å^3^)	1839.58(8)	1868.44(8)	1879.07(11)	1923.3(2)	1962.48(12)
*Z*	24				
μ (mm^–1^)	53.7	54.8	30.5	57.7	59.6
radiation	Mo Kα (λ = 0.71073)	Mo Kα (λ = 0.71073)	Ag Kα (λ = 0.56087)	Mo Kα (λ = 0.71073)	Mo Kα (λ = 0.71073)
*R*_int_	0.0550	0.0373	0.0540	0.0754	0.0565
final *R* indexes [*I* ≥ 2σ(*I*)]	*R*_1_/w*R*_2_ = 0.0410/0.0927	*R*_1_/w*R*_2_ = 0.0402/0.1062	*R*_1_/w*R*_2_ = 0.0301/0.0690	*R*_1_/w*R*_2_ = 0.0343/0.0437	*R*_1_/w*R*_2_ = 0.0263/0.0545
final *R* indexes [all data]	*R*_1_/w*R*_2_ = 0.0490/0.0950	*R*_1_/w*R*_2_ = 0.0513/0.1098	*R*_1_/w*R*_2_ = 0.0357/0.0712	*R*_1_/w*R*_2_ = 0.0630/0.0485	*R*_1_/w*R*_2_ = 0.0369/0.0575
largest diff. peak/hole (eÅ^–3^)	5.13/–4.36	3.78/–3.15	3.60/–3.56	1.79/–1.80	3.55/–2.00

a The complete table can be found in Supporting Information Chapter 1.

b Redetermination at 123 K of KTl.^9^

### 3.3 Partial Substitution of Cesium by Rubidium

The different mixtures of cesium and rubidium lead mainly to the formation of Cs_8–*x*_Rb*_x_*Tl_11_ phases and an alkali metal. Contrary to the potassium–rubidium mixture, no solid solution in the CsTl structure type was found. Instead, the new compounds Cs_1–*x*_Rb*_x_*Tl (*x* = 0.18, 0.42), which crystallize in the monoclinic space group *C*2/*c*, could be isolated from the mixture. The compound with 42% rubidium forms in samples with a rubidium proportion of 33–50%. In contrast, the one with 18% rubidium only forms with a high excess of both cesium and rubidium (see crystallographic data in Table 2; for PXRD patterns, atomic coordinates, and displacement parameters, see Supporting Information Chapters 1, 8, and 9). Measurements of crystals from different approaches did not show statistically significant deviations in composition. Quenching to room temperature from elevated temperatures is important to obtain the desired compounds, but phase purity still could not be obtained, which is in agreement with the reported binaries KTl and CsTl. This finding was supported by DSC and temperature-dependent PXRD (see Supporting Information Chapters 8.4and 8.5).

**Table 2:** Shortened Crystallographic Data of the Redetermined CsTl at 123 K and the New Ternary Compounds Cs_1–*x*_Rb_*x*_Tl (*x* = 0.18 (II), 0.42 (I))^a^

Empirical Formula	CsTl^b^	Cs_0.58_Rb_0.42_Tl (I)	Cs_0.82_Rb_0.18_Tl (II)
CSD number	2295021	2295125	2386360
formula weight	337.28	317.276	328.96
temperature (K)	123		
crystal system	orthorhombic	monoclinic	
space group	*Fddd*	*C*2/*c*	
*a* (Å)	9.1733(5)	14.2610(3)	14.4136(4)
*b* (Å)	15.0522(7)	11.1116(2)	11.1678(3)
*c* (Å)	31.8527(11)	27.5589(7)	40.8013(11)
β (°)	90	104.056(2)	96.353
volume (Å^3^)	4398.2(3)	4236.30(17)	6527.4(3)
z	48		72
μ (mm^–1^)	53.6	30.8	54.7
radiation	Mo Kα (λ = 0.71073)	Ag Kα (λ = 0.56087)	Mo Kα (λ = 0.71073)
*R*_int_	0.0430	0.0387	0.0867
final *R* indexes [*I* ≥ 2σ(*I*)]	*R*_1_/w*R*_2_ = 0.0466/0.1116	*R*_1_/w*R*_2_ = 0.0435/0.0868	*R*_1_/w*R*_2_ = 0.0600/0.1158
final *R* indexes [all data]	*R*_1_/w*R*_2_ = 0.0641/0.1229	*R*_1_/w*R*_2_ = 0.0634/0.0950	*R*_1_/w*R*_2_ = 0.0925/0.1243
largest diff. peak/hole (eÅ^–3^)	4.37/–4.57	4.36/–4.03	5.51/–2.89

a The complete table can be found in the Supporting Information Chapter 1.

b Redetermination at 123 K of CsTl.^10^

## 4 Discussion

### 4.1 Occupation Trends of the Alkali Metal Positions in K_1–*x*_Rb*_x_*Tl (*x* ≤ 0.69)

**Figure 2:**
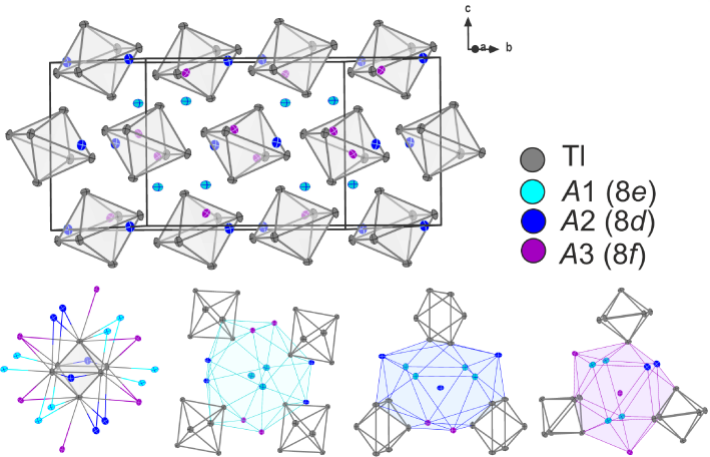
Unit cell, cluster coordination, and alkali metal coordination of structure type KTl.

Ternary materials according to K_1–*x*_Rb*_x_*Tl (*x* = 0–0.69) crystallize in the orthorhombic space group *Cmce* in the KTl structure type and contain compressed [Tl_6_]^6–^ octahedra as the thallium substructure. These are built by two symmetry-independent thallium atoms located at special positions (Tl1 Wyckoff 16g, Tl2 Wyckoff 8f). In addition, alkali metals occupy three crystallographically independent special sites (Wyckoff 8e, 8d, and 8f) (see Figure 2).

Potassium atoms at these three positions can be substituted partially by rubidium, while the rubidium proportion depends on the coordination number (CN) of the respective alkali metal position.

While the alkali metal position *A*3 (Wyckoff 8f, CN = 15) always shows the lowest rubidium content, position *A*1 (Wyckoff 8e, CN = 16) exhibits the highest site occupancy factors (s.o.f) for rubidium (see Table 3). This is also reflected in the formation energy calculations of ternaries K_0.67_Rb_0.33_Tl with rubidium being located on *A*1, *A*2, or *A*3 (see Section 5.4).

**Table 3:** Site Occupancy Factors (s.o.f) of the Symmetry-Independent Alkali Metal Positions of the Solid Solution in the KTl Structure Type^a^

Composition	s.o.f *A*1 (8e)		s.o.f *A*2 (8d)		s.o.f *A*3 (8f)	
KTl^b^	K	1	K	1	K	1
K_0.86_Rb_0.14_Tl	K Rb	0.782(13) 0.218	K Rb	0.881(13) 0.119	K Rb	0.930(13) 0.070
K_0.72_Rb_0.28_Tl	K Rb	0.623(7) 0.377	K Rb	0.730(8) 0.270	K Rb	0.813(7) 0.187
K_0.54_Rb_0.46_Tl	K Rb	0.414(8) 0.586	K Rb	0.528(8) 0.472	K Rb	0.687(7) 0.313
K_0.31_Rb_0.69_Tl	K Rb	0.244(7) 0.756	K Rb	0.281(7) 0.719	K Rb	0.419(7) 0.581

a The complete table can be found in Supporting Information Chapter 1.

b Redetermination of KTl at 123 K.

### 4.2 Structure Description of Cs_1–*x*_Rb*_x_*Tl (*x* = 0.18 (II), 0.42(I))

**Figure 3:**
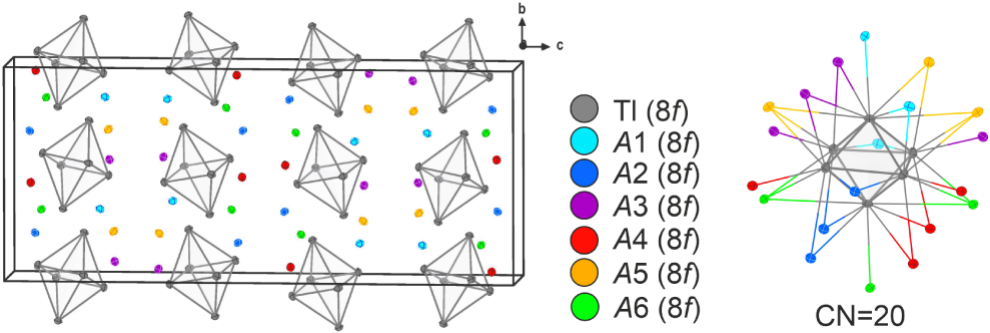
Unit cell and Tl-octahedron coordination sphere of (I).

The new compounds (I) and (II) crystallize in the monoclinic space group *C*2/*c*. Although there is a group-subgroup relationship between *C*2/*c* and *Cmce* (KTl) and *Fddd* (CsTl), respectively, the new compounds are not related in symmetry to the binary materials.^46−51^ Despite there is no crystallographic relationship between the new monoclinic compounds and KTl or CsTl, the structural relationship is defined by the type of thallium cluster and the similarity in the first coordination sphere of the different alkali metal positions (Figure 3 and Supporting InformationChapters 8 and 9).

The asymmetric unit of (I) consists of six symmetry-independent thallium positions and alkali metal positions, while in (II) there are nine symmetry-independent thallium and alkali metal positions, all of which are located on a general Wyckoff position of 8f. The compressed octahedra in the two new compounds are similar to those in CsTl,^10^ in the solid solution K_1–*x*_Rb*_x_*Tl, Cs_7.29_K_5.71_Tl_13_,^15^ A_10_Tl_6_O_2_ (A = K, Rb),^52^ Cs_10_Tl_6_TtO_4_ (Tt = Si, Ge), and Cs_10_Tl_6_SnO_3_^53^ (see the table with distances and distortion degree in Supporting Information Chapter 5).

**Figure 4:**
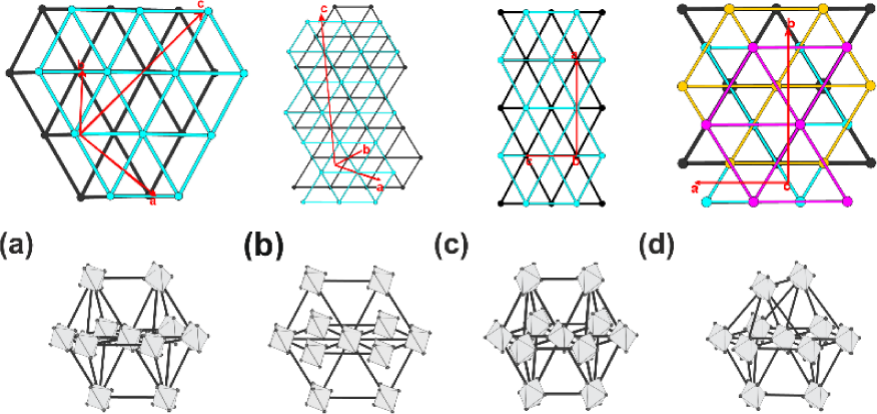
Cluster packing of (a) (I), (b) (II), (c) K_1–*x*_Rb*_x_*Tl, and (d) CsTl.

The [Tl_6_]^6–^ clusters of the compounds (I) and (II) arrange in AD hexagonal layers, which results in a distorted α-uranium packing.^54^ This is similar to KTl, whereas the clusters in CsTl pack in ADD’D’’ layers, which correspond to a distorted γ-plutonium packing (Figure 4).^55^

All hexathallide octahedra are each surrounded by 20 alkali metal atoms (*d* (Tl-*A*) ≤ 4.7 Å), six of which are exocoordinated to each vertex of the octahedron. Additionally, there are four face-capping and ten edge-capping alkali metal atoms (see Figure 3 and Supporting Information).

**Figure 5:**
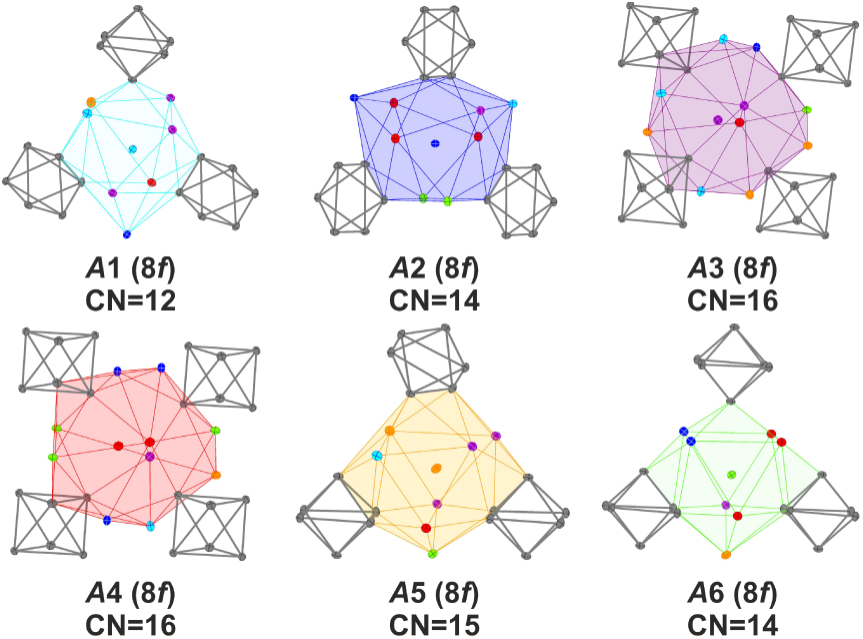
Alkali metal coordinations in (I).

All nine in (II) or six symmetry-independent alkali metal positions in (I) are mixed-occupied by cesium and rubidium. In the case of (I), the coordination numbers vary from 12, 14, and 15 to 16 (*d*(*A*-*A*) ≤ 5.5 Å; *d*(*A*-Tl) ≤ 4.51 Å) (see Figure 5). They can be differentiated, first of all, in coordination with three (*A*1, *A*2, *A*5, *A*6) and four [Tl_6_]^6—^ octahedra (*A*3, *A*4). The latter positions also show the highest cesium content (Table 4). In general, the coordination polyhedra are similar to those found in binary KTl and CsTl (see Figures 2, 5, and Supporting Information Chapter 6). The alkali metal positions *A*1, *A*5, and *A*6 are coordinated similarly to those of Cs3 (Wyckoff 16f) in CsTl. The ternary materials, when mixing rubidium and cesium, combine structural features of KTl and CsTl. As demonstrated in Figure 5, the coordination sphere of *A*2 is analogous to those of K2 (Wyckoff 8d) in KTl and Cs2 (Wyckoff 16g) in CsTl. However, it is important to note that positions *A*3 and *A*4 are surrounded like K1 (Wyckoff 8e) in KTl and Cs1 (Wyckoff 16g) in CsTl.

**Table 4:** Site Occupancy Factors (s.o.f.) of the Symmetry Independent Alkali Metal Positions in Cs_1–*x*_Rb*_x_*Tl (*x* = 0.18, 0.42)

Alkali metal position	Cs_0.58_Rb_0.42_Tl (I)				Cs_0.82_Rb_0.18_Tl (II)			
*A*1	Rb	0.811(8)	Cs	0.189(8)	Rb	0.07 (17)	Cs	0.930(17)
*A*2	Rb	0.419(8)	Cs	0.581(8)	Rb	0.284(16)	Cs	0.716(16)
*A*3	Rb	0.237(9)	Cs	0.763(9)	Rb	0.120(17)	Cs	0.880(17)
*A*4	Rb	0.162(9)	Cs	0.838(9)	Rb	0.161(17)	Cs	0.839(17)
*A*5	Rb	0.256(9)	Cs	0.744(9)	Rb	0.078(17)	Cs	0.922(17)
*A*6	Rb	0.641(8)	Cs	0.359(8)	Rb	0.218(17)	Cs	0.782(17)
*A*7					Rb	0.104(17)	Cs	0.896(17)
*A*8					Rb	0.423(17)	Cs	0.577(17)
*A*9					Rb	0.120(17)	Cs	0.880(17)

In (II), the coordination numbers of the first coordination sphere of the alkali metal positions vary from 13, 14, to 16, and especially the surroundings of *A*3 and *A*8 show differences from the ones in (I) or CsTl, which may be the reason for forming a different structure type (see Supporting Information Chapter 9).

The site occupancy factors for *A*1 to *A*6 (in the case of (I)) or *A*9 (in the case of (II)) show a general trend: the higher the coordination number, the higher the cesium content. This results in the sequence for decreasing cesium content and increasing rubidium content in (I), *A*4 > *A*3 > *A*5 > *A*2 > *A*6 > *A*1 (see Table 4).

## 5 DFT Results and Discussion

To support the experimental findings, theoretical calculations were applied in order to gain deeper insights into the structural stability of the reported compounds. The first issue addressed was the general question of the stability of binary and ternary compounds in the KTl and CsTl structure types when rubidium is involved.

### 5.1 Formation Energy

**Figure 6:**
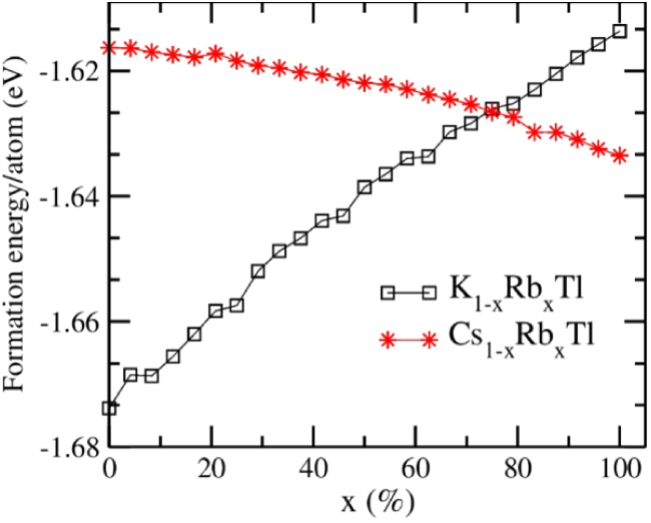
Calculated formation energy vs rubidium concentration in the KTl (black) and CsTl structure types (red).

In DFT, the formation energy is crucial for assessing the stability of atomic substitutions in crystal structures and chemical reactions. The formation energies of K_1–*x*_Rb*_x_*Tl and Cs_1–*x*_Rb*_x_*Tl were calculated according to eqs 1 and 2:
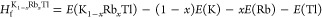
1
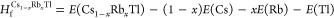
2where *E*((K/Cs)_1–*x*_Rb*_x_*Tl) is the total ground-state energy of (K/Cs)_1–*x*_Rb*_x_*Tl and *E*(K/Cs), *E*(Rb), and *E*(Tl) are the total ground-state energies of individual K/Cs, Rb, and Tl atoms, respectively, in their standard configurations. The calculated formation energy of K_1–*x*_Rb*_x_*Tl and Cs_1–*x*_Rb*_x_*Tl per atom is shown in Figure 6. The increasing rubidium concentration in KTl shows an increasing trend in the formation energy as it approaches RbTl. This is due to the larger size of rubidium compared to potassium, whereas a higher rubidium concentration in CsTl shows a decreasing trend due to the smaller size of rubidium compared to cesium.

### 5.2 Phonon Frequency Calculations

**Figure 7:**
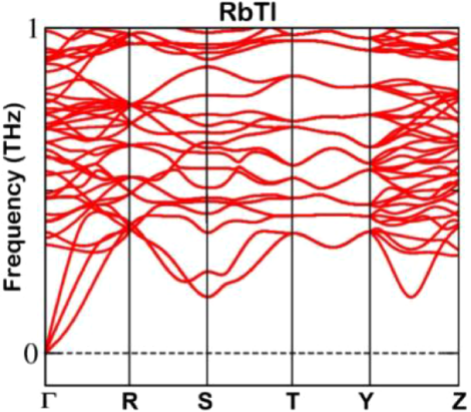
Phonon band structure of RbTl in the KTl structure type.

The aim of the formation energy calculations was to check the trend in both structure types with increasing rubidium concentration; however, claiming the structural stability with respect to the formation energy is not reliable. Therefore, phonon frequencies were used as indicators to investigate the dynamical stability of RbTl. The presence of positive phonon frequencies in a material is an indicator of its dynamic stability, and vice versa. In Figure 7, the calculated acoustic branches at gamma (Γ) show that there are no negative frequencies involved.

### 5.3 Electronic Structure Density of States

**Figure 8:**
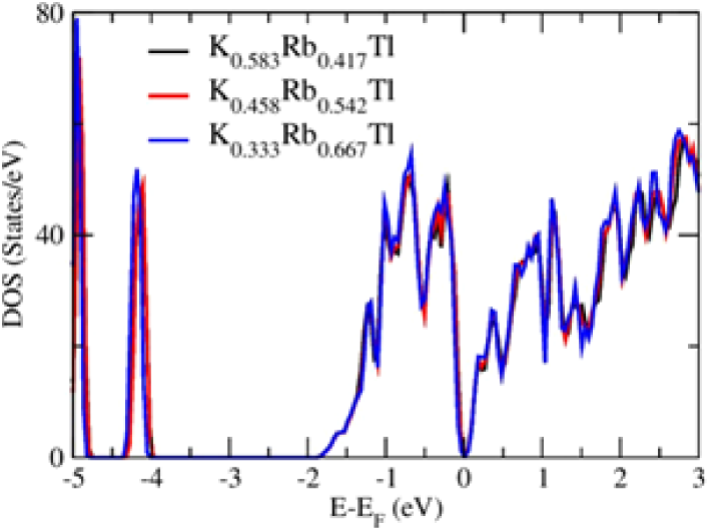
Calculated density of states of various rubidium concentrations in the KTl structure type.

The density of states (DOS) plays a key role in understanding materials’ electronic structure, predicting their macroscopic physical properties, and their response to various external conditions such as temperature and pressure. In this work, the DOS for three different concentrations of rubidium in the KTl structure type were calculated to observe the effect of the substitution, as shown in Figure 8. The calculated DOS for three different concentrations show good agreement, and no significant difference is observed between them. This clarifies that the electronic structure of K_1–*x*_Rb*_x_*Tl (*x* > 0) is similar to KTl, and the identifiable difference is due to the size effect or the different ionic radii of rubidium.

**Figure 9:**
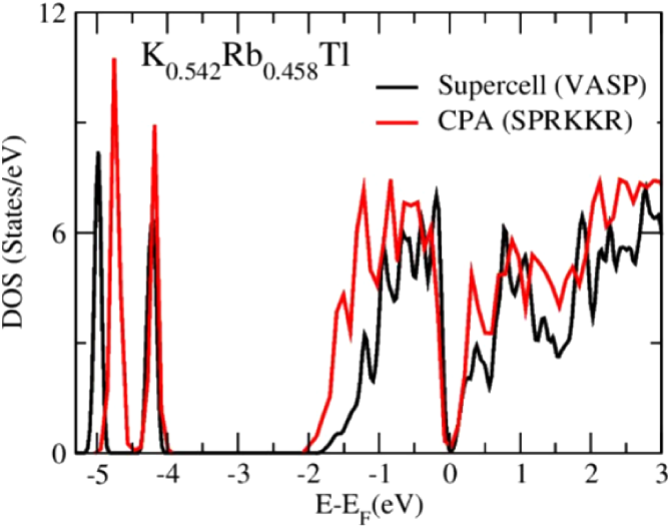
Calculated total density of states (TDOS) using the VASP and SPRKKR codes.

Figure 9 shows the calculated total density of states (TDOS) by using the supercell approach and the CPA implemented in the VASP and SPRKKR codes. The CPA enables the analysis of mixed occupancy of crystallographic positions without needing a fully ordered supercell model, effectively representing the statistically disordered crystal structure.^40,56^ In the energy range from −2.0 to 0.0 eV, the increasing bandwidth from −1.8 to −2.0 eV and the shifting of the low-energy peak (around −5 eV) can, therefore, be attributed to the disorder effect in terms of the impact of either atomic randomness or partial order of different atoms at the same site. In contrast, the electronic structure around the Fermi level is similar (more detailed description and atomic coordinates of the ordered model in Supporting Information Chapter 10).

### 5.4 Stability of Rubidium at Different Sites in K_0.67_Rb_0.33_Tl

**Figure 10:**
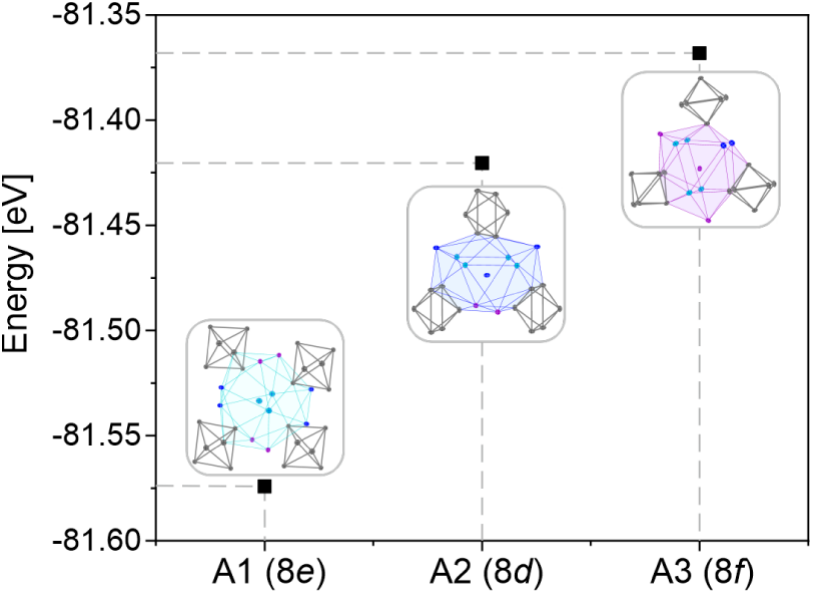
Ground state calculations for K_0.67_Rb_0.33_Tl in the KTl structure type, while rubidium is located on the three different alkali metal positions *A*1–*A*3. A detailed discussion of the alkali metal sites can be found in Section 4.1.

To check the stability of different configurations of rubidium on the three crystallographically different alkali metal positions (see Figure 2) according to K_0.67_Rb_0.33_Tl, the ground-state energies for three hypothetical situations were calculated (see Figure 10 and Supporting Information Chapter 11). This clearly shows that the favored position for rubidium is *A*1. This result is in excellent agreement with the observations of the standard error of the effect from the experimental data (see Table 3).

## 6 Conclusions

In-depth experimental investigations did not allow for the preparation of a binary RbTl. Mixed alkali metal approaches K_1–*x*_Rb_*x*_Tl suggest that the KTl structure type is favored up to *x* = 0.69, while the CsTl type could not be realized in ternary approaches. In addition, in these cases, the formation of lower-symmetry monoclinic compounds underlines this experimental finding. The DFT-calculated formation energies were used to investigate the trend between the two phases with an increasing rubidium concentration. At the same time, phonon calculations were used as an indicator of dynamic stability in hypothetical RbTl. The electronic structure calculation of rubidium in the KTl structure type suggests that an increasing rubidium content does not affect the electronic structure around the Fermi level. The preference for rubidium on the *A*1 crystallographic site is observed in experiments and is also independently obtained in the calculated ground-state energies. The discrepancy between the theoretically dynamically stable but experimentally unobservable binary RbTl might be explained by the effect of temperature. Future experiments will show if the application of more sophisticated cooling and quenching techniques could facilitate higher concentrations of rubidium in A_1–*x*_Rb_*x*_ Tl (A= K, Cs) compounds.

## Supporting Information Available

Experimental and DFT calculation details, crystallographic data, additional structure description, and powder diffraction patterns (PDF)
