# Supplementary material for: Approaching Hypothetical RbTl in Experiments and Theory – X-ray Structure Determination of Cs1–xRbxTl (x = 0.18, 0.42) and a Solid Solution K1–xRbxTl (x ≤ 0.69)
Source: Inorg Chem. 2025 Apr 3;64(14):6879–87. doi: 10.1021/acs.inorgchem.4c05305 (PMC12001245; doi:10.1021/acs.inorgchem.4c05305)
Supplement: Supplementary file 1 — ic4c05305_si_001.pdf [file ic4c05305_si_001.pdf]

Supplementary Information

Approaching Hypothetical RbTl in Experiment and Theory - X-Ray Structure Determination of $Cs_{1-x}Rb_xTl$ (x=0.18, 0.42) and a Solid Solution $K_{1-x}Rb_xTl$ (x≤0.69)

Vanessa F. Schwinghammer,[a,b] Saleem A. Khan,[c] Susanne M. Tiefenthaler,[a] Tomáš Kovářík,[c] Ján Minár,[c] and Stefanie Gärtner* [a,b]

a. University of Regensburg, Department of Inorganic Chemistry, Universitätsstraße 31, 93053 Regensburg, Germany

b. University of Regensburg, Central Analytics X-Ray, Universitätsstraße 31, 93053 Regensburg, Germany

c. New Technologies-Research Center, University of West Bohemia, 30100 Pilsen, Czech Republic

*Corresponding Author: Stefanie.Gaertner@ur.de

Content

1. **Crystallographic Data** 4
2. **Solid-State Approaches** 6
   - **2.1 Powder Diffraction Pattern of the Sample RbTl** 8
   - **2.2 Powder Diffraction Pattern of the Sample $Rb_{1.1}Tl$** 8
   - **2.3 Powder Diffraction Pattern of the Sample KTl** 9
   - **2.4 Powder Diffraction Pattern of the Sample K_5RbTl_6** 9
   - **2.5 Powder Diffraction Pattern of the Sample K_2RbTl_3** 10
   - **2.6 Powder Diffraction Pattern of the Sample $KRbTl_2$ (Temp. 2)** 10
   - **2.7 Powder Diffraction Pattern of the Sample $KRbTl_2$ (Temp. 3)** 11
   - **2.8 Powder Diffraction Pattern of the Sample KRb_2Tl_3 (Temp. 3)** 11
   - **2.9 Powder Diffraction Pattern of the Sample KRb_5Tl_6 (Temp. 5)** 12
   - **2.10 Powder Diffraction Pattern of the Sample $K_5Rb_5Tl_7$** 12
   - **2.11 Powder Diffraction Pattern of the Sample $K_3Rb_7Tl_7$** 13
   - **2.12 Powder Diffraction Pattern of the Sample CsTl** 13
   - **2.13 Powder Diffraction Pattern of the Sample Cs_5RbTl_6** 14
   - **2.14 Powder Diffraction Pattern of the Sample Cs_2RbTl_3 (Temp. 6)** 14
   - **2.15 Powder Diffraction Pattern of the Sample $CsRbTl_2$** 15
   - **2.16 Powder Diffraction Pattern of the Sample $Cs_6Rb_4Tl_{10}$** 15
   - **2.18 Powder Diffraction Pattern of the Sample $CsRb_2Tl_3$** 17
   - **2.19 Powder Diffraction Pattern of the Sample $CsRb_5Tl_6$ (Temp. 6)** 17
3. **SEM/EDS Measurements** 18
   - **3.1 Sample Cs_5RbTl_6** 18
   - **3.2 Measurements on the Approach K_5RbTl_6** 20
4. **Direct Reduction Experiments in Liquid Ammonia** 21
   - **4.1 $TlBF_4$ + 2 Rb + NH_3(l)** 21
   - **4.2 $TlPF_6$ + 2 Rb + NH_3(l)** 21
   - **4.3 TlBr + 2 Rb + NH_3(l)** 22
5. **Distances of Compressed $[Tl_6]^{6-}$ Octahedra** 23
6. **CsTl** 24
7. **Trend of the Solid Solution $K_{1-x}Rb_xTl$ (x≤0.69)** 25
8. **$Cs_{0.58}Rb_{0.42}Tl$** 26
   - **8.1 Atomic Coordinates** 26
   - **8.2 Displacement Parameter** 27
   - **8.3 Distances** 28
   - **8.4 DSC Measurements** 29

8.5 Temperature Dependent PXRD 30
9. $Cs_{0.82}Rb_{0.18}Tl$ 32
9.1 Structure Description 32
9.2 Atomic Coordinates 34
9.3 Displacement Parameters 35
9.4 Distances 36
10 Effect of Disorder on the Electronic Structure of $K_{0.542}Rb_{0.458}Tl$ 38
11 Ground State Formation Energy Calculations of $K_{0.667}Rb_{0.333}Tl$ 39
12 Details on Phonon Calculations 39
References 40

1. Crystallographic Data

Table S1: Crystallographic data of the different compositions in the solid solution $K_{1-x}Rb_xTl$ ($x \leq 0.69$) including the redetermination of KTl.[1]

Empirical Formula	KTl*	$K_{0.86}Rb_{0.14}Tl$	$K_{0.72}Rb_{0.28}Tl$	$K_{0.54}Rb_{0.46}Tl$	$K_{0.31}Rb_{0.69}Tl$
CSD number	2295143	2295126	2295113	2295115	2348700
Formula weight	243.482	249.87	256.36	264.66	275.25
Temperature (K)	123				
Crystal system	Orthorhombic				
Space group	*Cmce*				
a (Å)	15.2382(4)	15.3150(3)	15.3508(4)	15.4681(11)	15.5750(6)
b (Å)	14.9476(4)	15.0130(3)	15.0226(5)	15.1269(9)	15.2312(5)
c (Å)	8.0763(2)	8.1263(2)	8.1483(3)	8.2196(5)	8.2726(3)
Volume (Å³)	1839.58(8)	1868.44(8)	1879.07(11)	1923.3(2)	1962.48(12)
Z	24				
ρ_{calc} (g/cm³)	5.28	5.33	5.44	5.48	5.59
μ (mm^{-1})	53.7	54.8	30.5	57.7	59.6
F(000)	2355.6	2460.0	2520.0	2597.0	2696.0
Crystal size (mm³)	0.09 x 0.05 x 0.02	0.06 x 0.05 x 0.04	0.076 x 0.05 x 0.02	0.093 x 0.088 x 0.07	0.12 x 0.028 x 0.023
Radiation	Mo Kα (λ=0.71073)	Mo Kα (λ=0.71073)	Ag Kα (λ=0.56087)	Mo Kα (λ=0.71073)	Mo Kα (λ=0.71073)
2Θ range for data collection (°)	5.34 to 77.14	5.32 to 80.458	4.28 to 58.934	6.226 to 66.282	5.23 to 71.26
Index ranges	-26≤h≤22 -21≤k≤26 -14≤l≤13	-25≤h≤27 -26≤k≤27 -10≤l≤14	-26≤h≤26 -23≤k≤26 -13≤l≤14	-23≤h≤21 -23≤k≤20 -12≤l≤12	-25≤h≤24 -24≤k≤24 -12≤l≤13
Reflections independent/ collected	2675/19253	2985/30940	2739/19623	1894/11220	2340/27443
Data/ restraints/ parameter	2675/0/32	2985/0/32	2739/0/35	1894/0/35	2340/0/35
Goodness-of-fit on F²	1.018	1.091	1.130	1.032	1.021
R_{int}	0.0550	0.0373	0.0540	0.0754	0.0565
Final R indexes [$I \geq 2\sigma(I)$]	R_1/wR_2= 0.0410/0.0927	R_1/wR_2= 0.0402/0.1062	R_1/wR_2= 0.0301/0.0690	R_1/wR_2= 0.0343/0.0437	R_1/wR_2=0.0263/0.0545
Final R indexes [all data]	R_1/wR_2= 0.0490/0.0950	R_1/wR_2= 0.0513/0.1098	R_1/wR_2= 0.0357/0.0712	R_1/wR_2= 0.0630/0.0485	R_1/wR_2=0.0369/0.0575
Largest diff. peak/hole (e$Å^{-3}$)	5.13/-4.36	3.78/-3.15	3.60/-3.56	1.79/-1.80	3.55/-2.00

*Redetermination at 123 K of KTl from *Corbett et al.*[1]

Table S2: Crystallographic data of the compounds $Cs_{1-x}Rb_xTl$ (x=0.18, 0.42) and the redetermination of CsTl[2].

Empirical Formula	CsTl*	$Cs_{0.58}Rb_{0.42}Tl$	$Cs_{0.82}Rb_{0.18}Tl$
CSD number	2295021	2295125	2386360
Formula weight	337.28	317.276	328.96
Temperature (K)		123	
Crystal system	orthorhombic	Monoclinic	
Space group	*Fddd*	*C2/c*	
a (Å)	9.1733(5)	14.2610(3)	14.4136(4)
b (Å)	15.0522(7)	11.1116(2)	11.1678(3)
c (Å)	31.8527(11)	27.5589(7)	40.8013(11)
β (°)	90	104.056(2)	96.353
Volume (Å³)	4398.2(3)	4236.30(17)	6527.4(3)
Z	48		72
ρ_{calc} (g/cm³)	6.11	5.97	6.03
μ (mm^{-1})	53.6	30.8	54.7
F(000)	6528.0	6115.6	9565.0
Crystal size (mm³)	0.123 x 0.104 x 0.077	0.115 x 0.07 x 0.05	0.074 x 0.065 x 0.041
Radiation	Mo Kα (λ=0.71073)	Ag Kα (λ=0.56087)	Mo Kα (λ=0.71073)
2Θ range for data collection (°)	8.246 to 71.03	4.68 to 61.3	4.624 to 64.064
Index ranges	-14≤h≤13<br>-24≤k≤14<br>-41≤l≤52	-25≤h≤25<br>-20≤k≤20<br>-49≤l≤29	-18≤h≤21<br>-16≤k≤16<br>-60≤l≤59
Reflections independent/ collected	2391/6633	12520/34231	11178/58874
Data/ restraints/ parameter	2392/0/30	12520/0/115	11178/0/191
Goodness-of-fit on F^2	1.073	1.048	1.136
R_{int}	0.0430	0.0387	0.0867
Final R indexes [$I \geq 2\sigma(I)$]	R_1/wR_2= 0.0466/0.1116	R_1/wR_2= 0.0435/0.0868	R_1/wR_2= 0.0600/0.1158
Final R indexes [all data]	R_1/wR_2= 0.0641/0.1229	R_1/wR_2= 0.0634/0.0950	R_1/wR_2= 0.0925/0.1243
Largest diff. peak/hole (eÅ^{-3})	4.37/-4.57	4.36/-4.03	5.51/-2.89

* Redetermination at 123 K of CsTl from *Corbett et al.*[2]

2. Solid-State Approaches

While the binary phase diagrams of the K-Tl, Rb-Tl, and Cs-Tl systems are long-time known, there is no information about ternary phase diagrams given so far.[3] First we tried to applied temperature program 2 (see below), which is based on the one from Dong & Corbett (KTl route (c)[1], CsTl[2]).

The K-Tl phase diagram shows a peritectic point for the 50:50 composition, so quenching should result in a higher yield of KTl, which also was observed by Dong & Corbett. Up to the approach $KRbTl_2$ the solid solution $K_{1-x}Rb_xTl$ can be obtained via slow cooling and quenching with the very stable side product $K_{8-x}Rb_xTl_{11}$. Stochiometric samples with a higher rubidium content than 50% mainly resulted in $K_{8-x}Rb_xTl_{11}$ and a sticky metallic substance regardless of the temperature program. Therefore, we used a high excess of alkali metal and quenching, to have enough alkali metal available in addition to the formation of the by-product to obtain $K_{1-x}Rb_xTl$ possibly. With this procedure, we were able to increase the rubidium content up to 69%. Comparing the result of the stoichiometric and excess samples with a 50:50 proportion of potassium and rubidium (2x $KRbTl_2$, $K_5Rb_5Tl_7$) shows, that quenching decreases the $K_{8-x}Rb_xTl_{11}$ content significantly. On the other hand annealing after quenching to room temperature does not show a great effect on the formed product.

In case of the Cs-Tl system, the phase diagram does not show the composition CsTl. From the published experimental results on this compound, it can be assumed that a peritectic point can also be expected with this composition due to the formation of Cs_8Tl_{11} after annealing.[2] CsTl can be obtained by slow cooling and quenching, because of that both temperature programs were applied, but from the experience gained from the first few examples and the K-Rb-Tl system, we focused on quenching. Samples with a rubidium content between 33% and 50% contain the new ternary compound $Cs_{0.58}Rb_{0.42}Tl$ next to the side product $Cs_{8-x}Rb_xTl_{11}$.

Temperature program 1 (based on the temperature program of $K_{10}Tl_7$)[4]

RT to 673 K hold for 48 h quenching in water annealing at 343 K for 14 days, at 323 K for seven days, and then cooling to RT with 5 K/h.

Temperature program 2

RT to 773 K, hold for 48 h, cooling to RT with 5 K/h.

Temperature program 3

RT to 773 K, hold for 48 h, quenching to RT in water.

Temperature program 4

RT to 773 K, hold for 48 h, quenching with liquid N_2.

Temperature program 5

RT to 773 K, hold for 48 h, cooling to 633 K with 100 K/h, hold for 48 h, quenching in water to RT.

Temperature program 6

RT to 673 K, hold for 48 h, quenching to RT in water.

Table S3: List of the different samples with the temperature program and results.

**Approach**	**Temperature Program**			**Result**			**Method**
		**Slow cooling**	**quenching**	**$A_{1-x}Rb_xTl$ (*A*=K, Cs)**	**$A_{8-x}Rb_xTl_{11}$ (*A*=K, Cs)**	**Other side product**	
RbTl	4		✓		✓	**$Rb_{15}Tl_{27}$**	PXRD, SCXRD
$Rb_{1.1}Tl$	4		✓		✓	**$Rb_{15}Tl_{27}$**	PXRD, SCXRD
KTl	2	✓		✓**(x=0)**	✓		PXRD, SCXRD
K_5RbTl_6	2	✓		✓**(x>0)**	✓		PXRD, SCXRD
K_2RbTl_3	2	✓		✓**(x>0)**	✓		PXRD, SCXRD
$KRbTl_2$	2	✓		✓**(x>0)**	✓		PXRD
$KRbTl_2$	3		✓	✓**(x>0)**			PXRD, SCXRD
KRb_2Tl_3	2	✓			✓		SCXRD
KRb_2Tl_3	3		✓	✓**(x>0)**	✓		PXRD, SCXRD
KRb_5Tl_6	2	✓			✓		SCXRD
KRb_5Tl_6	5		✓		✓		PXRD, SCXRD
KRb_5Tl_6	3		✓		✓		SCXRD
$K_5Rb_5Tl_7$	1		✓	✓**(x>0)**		**Rb**	PXRD, SCXRD
$K_3Rb_7Tl_7$	1		✓	✓**(x>0)**		**Rb**	PXRD, SCXRD
$K_{1.1}Rb_{2.1}Tl_3$	6		✓		✓		SCXRD
CsTl	2	✓		✓**(x=0)**	✓		PXRD, SCXRD
Cs_5RbTl_6	3		✓	✓**(x≈0)***	✓		PXRD, SCXRD
Cs_2RbTl_3	6		✓	✓**(x=0.42)**	✓		PXRD, SCXRD
Cs_2RbTl_3	2	✓			✓		SCXRD
$CsRbTl_2$	6		✓	✓**(x=0.42)**	✓		PXRD, SCXRD
$Cs_6Rb_4Tl_{10}$	4		✓	✓**(x=0.42)**	✓		PXRD, SCXRD
$Cs_{0.58}Rb_{0.42}Tl$	3		✓	✓**(x=0.42)**	✓		PXRD
$CsRb_2Tl_3$	3		✓		✓		SCXRD
$CsRb_5Tl_6$	2	✓			✓		SCXRD
$CsRb_5Tl_6$	6		✓	✓**(x≈0)***	✓		SCXRD, PXRD
$Cs_7Rb_3Tl_7$	1		✓	✓**(x=0.18)**		**Cs, Rb**	SCXRD

** Due to the accuracy of the method, the involvement of rubidium cannot be ruled out 100%, SCXRD showed hardly any deviation from the known cell parameter, and refinement of mixed occupancy did not yield a significant rubidium content.*

2.1 Powder Diffraction Pattern of the Sample RbTl

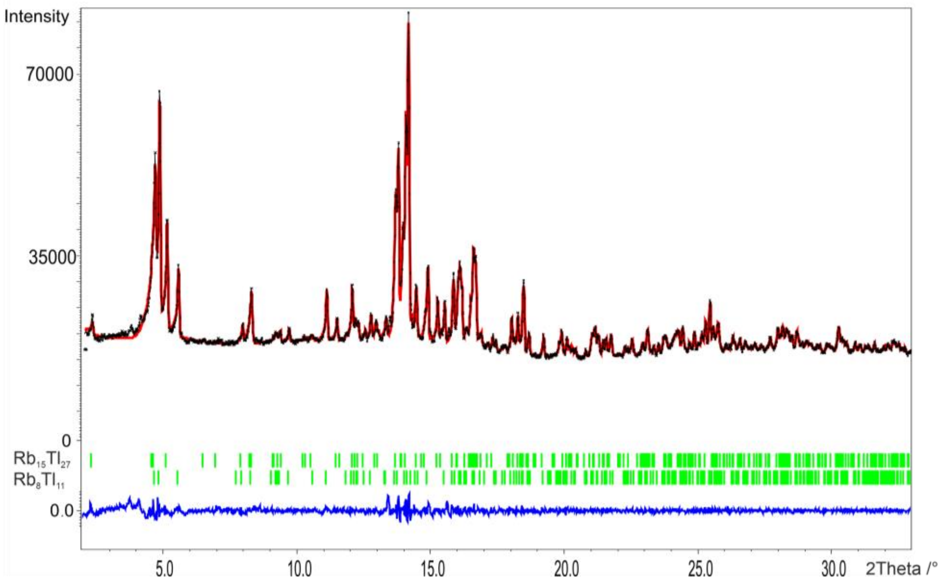

Figure S 1: Measured powder diffraction pattern of the approach RbTl (black). The refinement was carried out with the LeBail algorithm in *JANA2006*. The vertical bars (green) underneath the powder pattern show the calculated reflection positions. The curve at the bottom (blue) represents the difference plot. GOF=1.19, R_p=1.87, R_{wp}=2.66, x-axis: 2Theta in °, y-axis: Intensity.

2.2 Powder Diffraction Pattern of the Sample $Rb_{1.1}Tl$

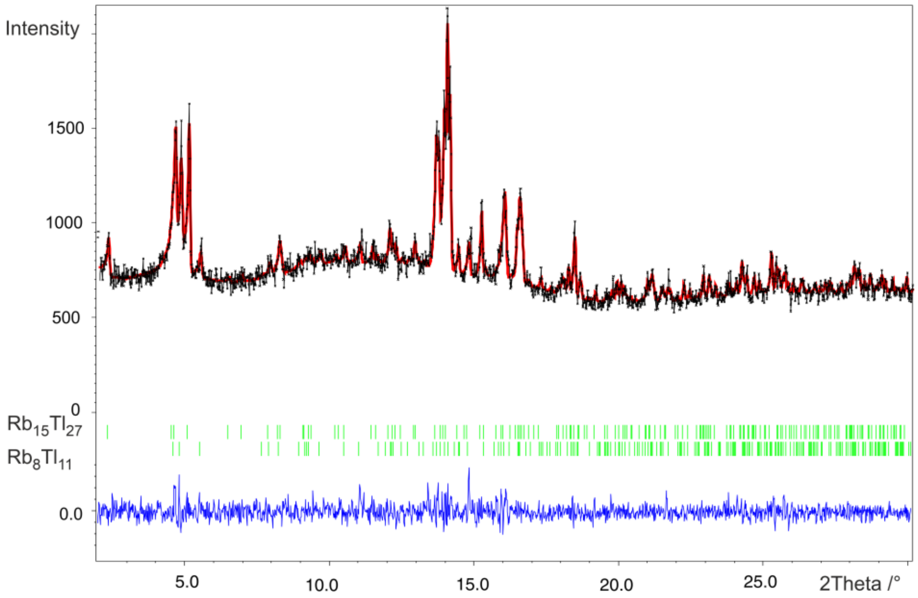

Figure S 2: Measured powder diffraction pattern of the approach $Rb_{1.1}Tl$ (black). The refinement was carried out with the LeBail algorithm in JANA2006. The vertical bars (green) underneath the powder pattern show the calculated reflection positions. The curve at the bottom (blue) represents the difference plot. GOF=0.86, R_p=2.12, R_{wp}=2.81, x-axis: 2Theta in °, y-axis: Intensity.

2.3 Powder Diffraction Pattern of the Sample KTl

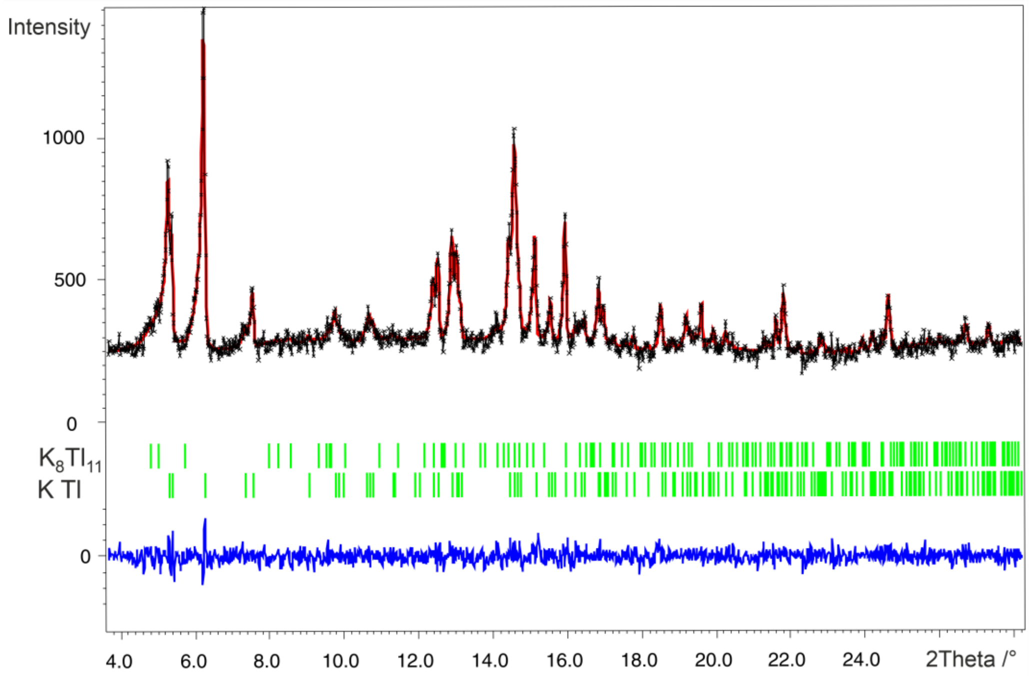

Figure S 3: Measured powder diffraction pattern of the approach KTl (black). The refinement was carried out with the LeBail algorithm in JANA2006. The vertical bars (green) underneath the powder pattern show the calculated reflection positions. The curve at the bottom (blue) represents the difference plot. GOF=1.04, R_p=3.55, R_{wp}=4.97, x-axis: 2Theta in °, y-axis: Intensity.

2.4 Powder Diffraction Pattern of the Sample K_5RbTl_6

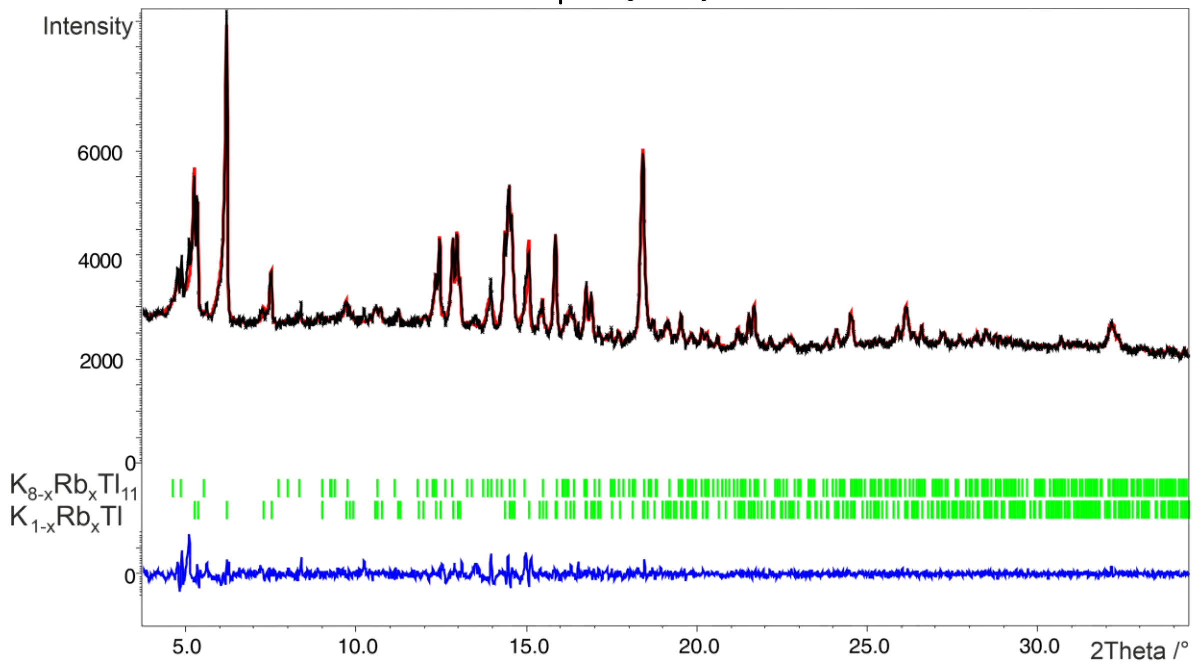

Figure S 4: Measured powder diffraction pattern of the approach K_5RbTl_6 (black). The refinement was carried out with the LeBail algorithm in JANA2006. The vertical bars (green) underneath the powder pattern show the calculated reflection positions. The curve at the bottom (blue) represents the difference plot. GOF=1.15, R_p=1.64, R_{wp}=2.32, x-axis: 2Theta in °, y-axis: Intensity.

2.5 Powder Diffraction Pattern of the Sample K_2RbTl_3

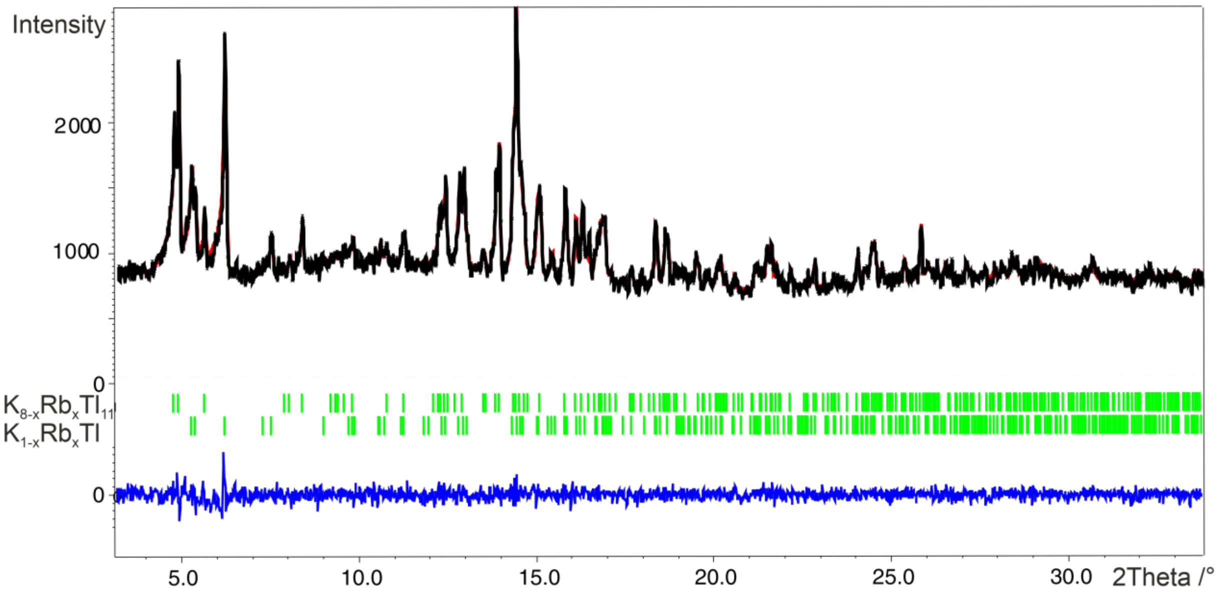

Figure S 5: Measured powder diffraction pattern of the approach K_2RbTl_3 (black). The refinement was carried out with the LeBail algorithm in JANA2006. The vertical bars (green) underneath the powder pattern show the calculated reflection positions. The curve at the bottom (blue) represents the difference plot. GOF=0.82, R_p=1.78, R_{wp}=2.35, x-axis: 2Theta in °, y-axis: Intensity.

2.6 Powder Diffraction Pattern of the Sample $KRbTl_2$ (Temp. 2)

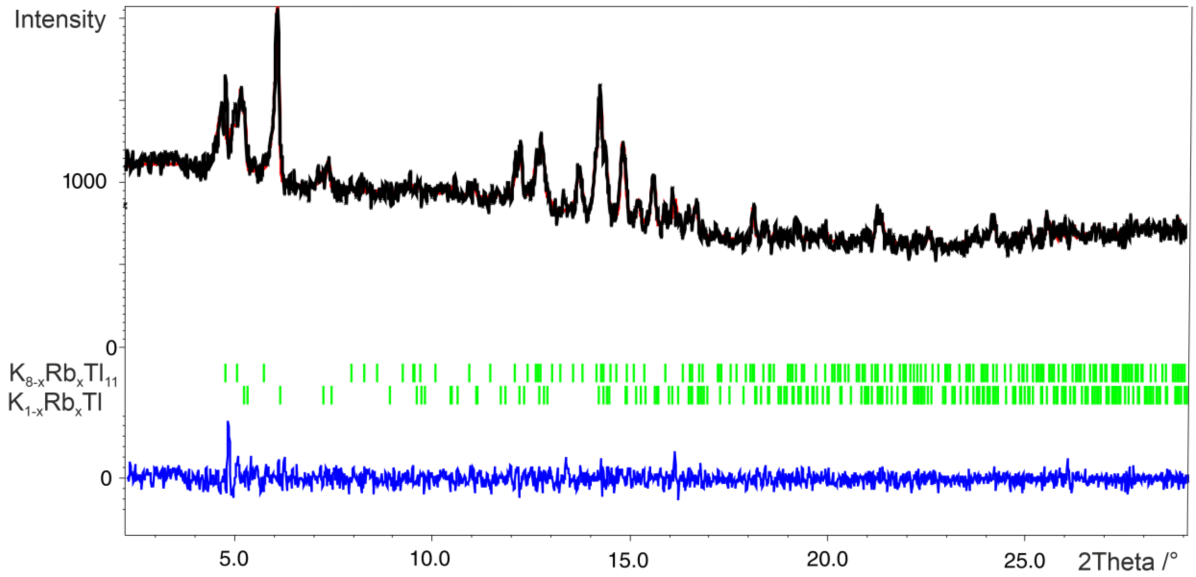

Figure S 6: Measured powder diffraction pattern of the approach $KRbTl_2$ (black). The refinement was carried out with the LeBail algorithm in JANA2006. The vertical bars (green) underneath the powder pattern show the calculated reflection positions. The curve at the bottom (blue) represents the difference plot. GOF=0.86, R_p=1.94, R_{wp}=2.56, x-axis: 2Theta in °, y-axis: Intensity.

2.7 Powder Diffraction Pattern of the Sample $KRbTl_2$ (Temp. 3)

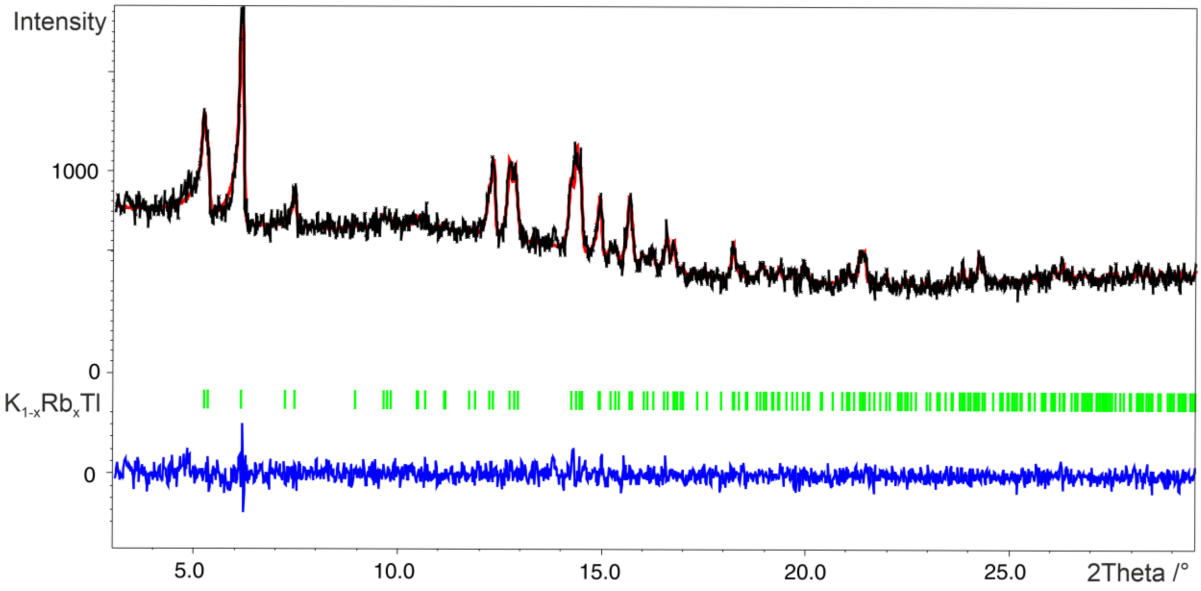

Figure S 7: Measured powder diffraction pattern of the approach $KRbTl_2$ (black). The refinement was carried out with the LeBail algorithm in JANA2006. The vertical bars (green) underneath the powder pattern show the calculated reflection positions. The curve at the bottom (blue) represents the difference plot. GOF=0.83, R_p=2.14, R_{wp}=2.77, x-axis: 2Theta in °, y-axis: Intensity.

2.8 Powder Diffraction Pattern of the Sample KRb_2Tl_3 (Temp. 3)

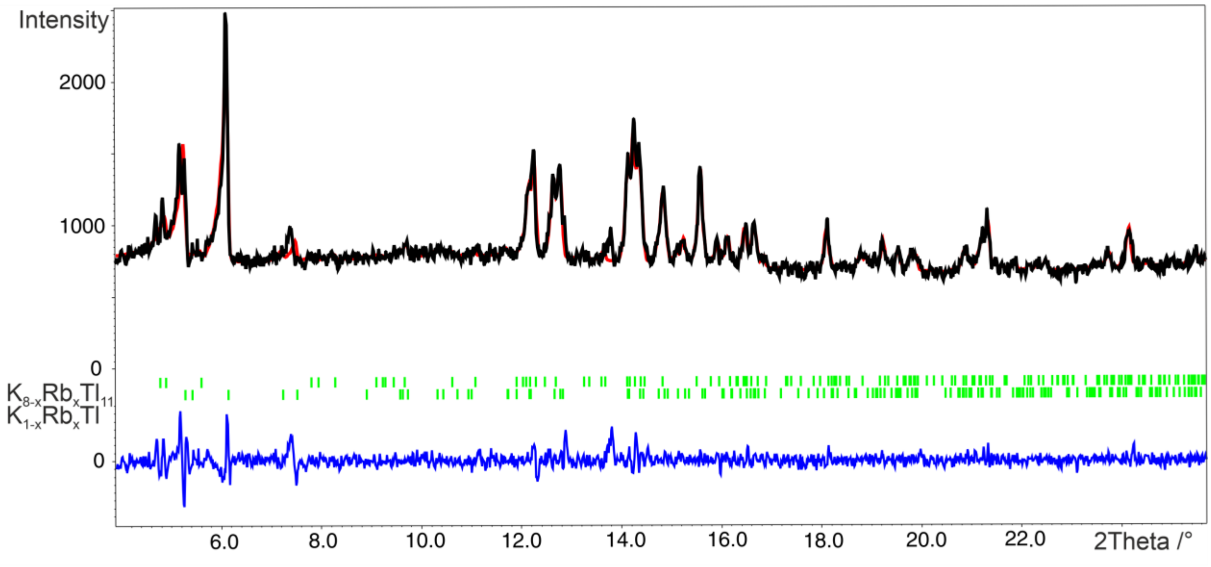

Figure S 8: Measured powder diffraction pattern of the approach KRb_2Tl_3 (black). The refinement was carried out with the LeBail algorithm in JANA2006. The vertical bars (green) underneath the powder pattern show the calculated reflection positions. The curve at the bottom (blue) represents the difference plot. GOF=0.98, R_p=2.17, R_{wp}=3.06, x-axis: 2Theta in °, y-axis: Intensity.

2.9 Powder Diffraction Pattern of the Sample KRb_5Tl_6 (Temp. 5)

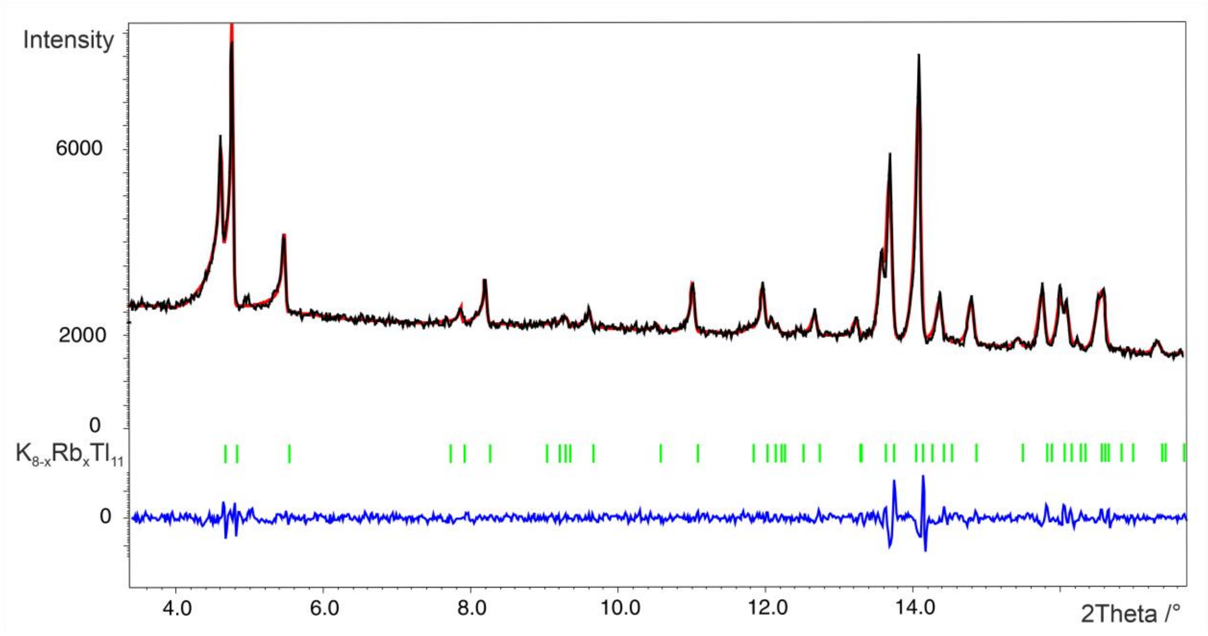

Figure S 9: Measured powder diffraction pattern of the approach KRb_5Tl_6 (black). The refinement was carried out with the LeBail algorithm in JANA2006. The vertical bars (green) underneath the powder pattern show the calculated reflection positions. The curve at the bottom (blue) represents the difference plot. GOF=1.39, R_p=1.80, R_{wp}=2.72, x-axis: 2Theta in °, y-axis: Intensity.

2.10 Powder Diffraction Pattern of the Sample $K_5Rb_5Tl_7$

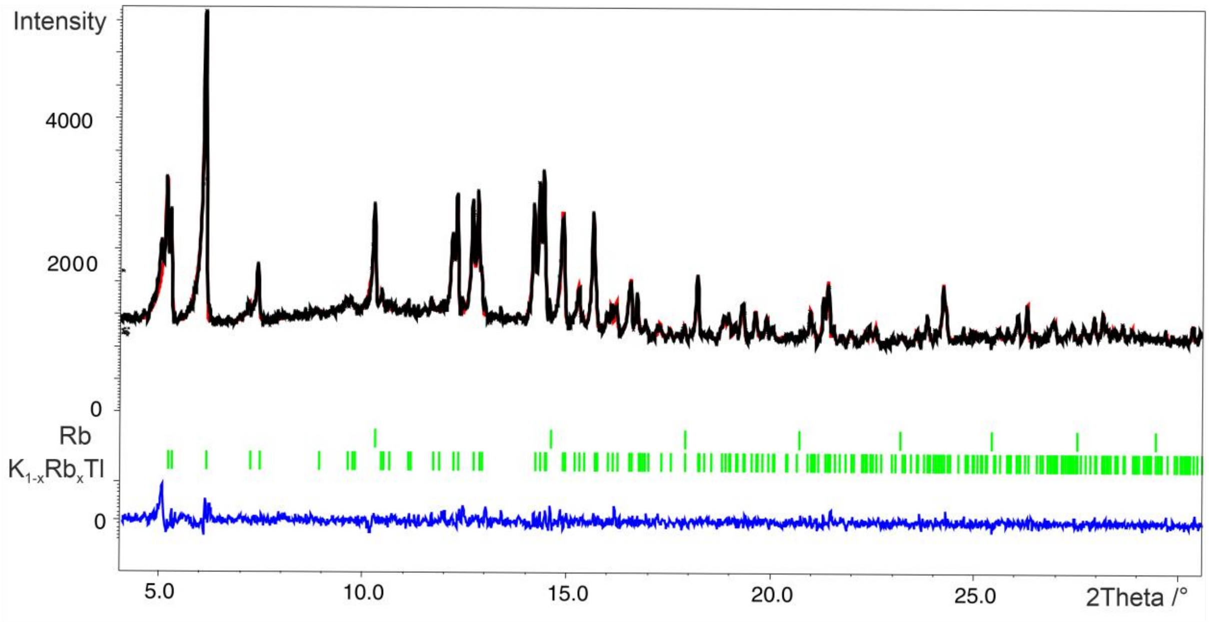

Figure S 10: Measured powder diffraction pattern of the approach $K_5Rb_5Tl_7$ (black). The refinement was carried out with the LeBail algorithm in JANA2006. The vertical bars (green) underneath the powder pattern show the calculated reflection positions. The curve at the bottom (blue) represents the difference plot. GOF=0.93, R_p=1.50, R_{wp}=2.02, x-axis: 2Theta in °, y-axis: Intensity.

2.11 Powder Diffraction Pattern of the Sample $K_3Rb_7Tl_7$

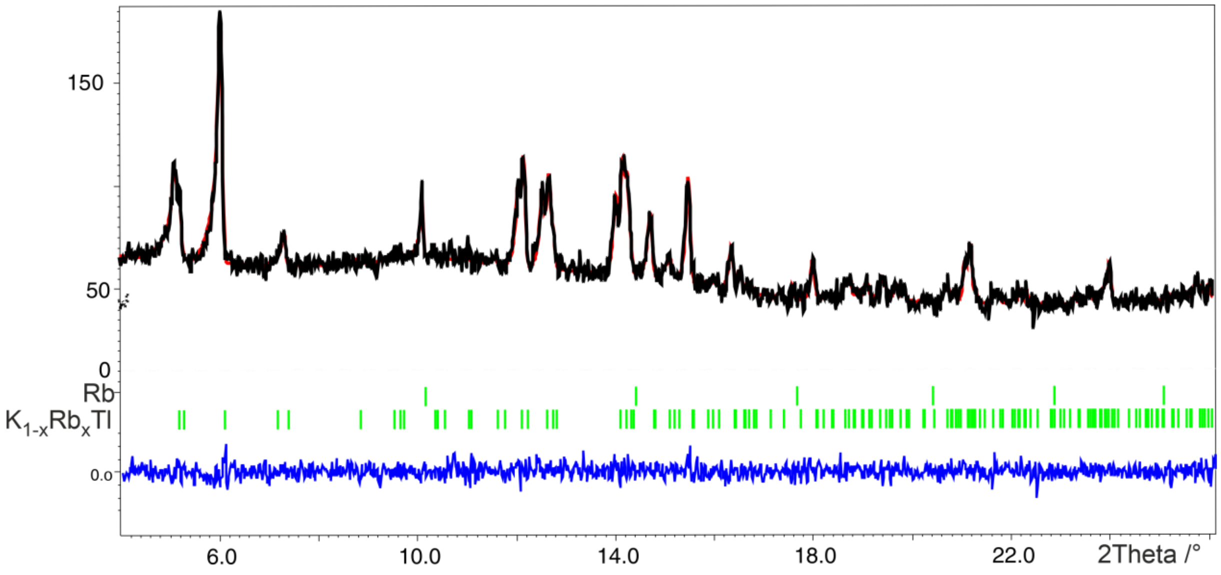

Figure S 11: Measured powder diffraction pattern of the approach $K_3Rb_7Tl_7$ (black). The refinement was carried out with the LeBail algorithm in JANA2006. The vertical bars (green) underneath the powder pattern show the calculated reflection positions. The curve at the bottom (blue) represents the difference plot. GOF=0.79, R_p=2.14, R_{wp}=2.74, x-axis: 2Theta in °, y-axis: Intensity.

2.12 Powder Diffraction Pattern of the Sample CsTl

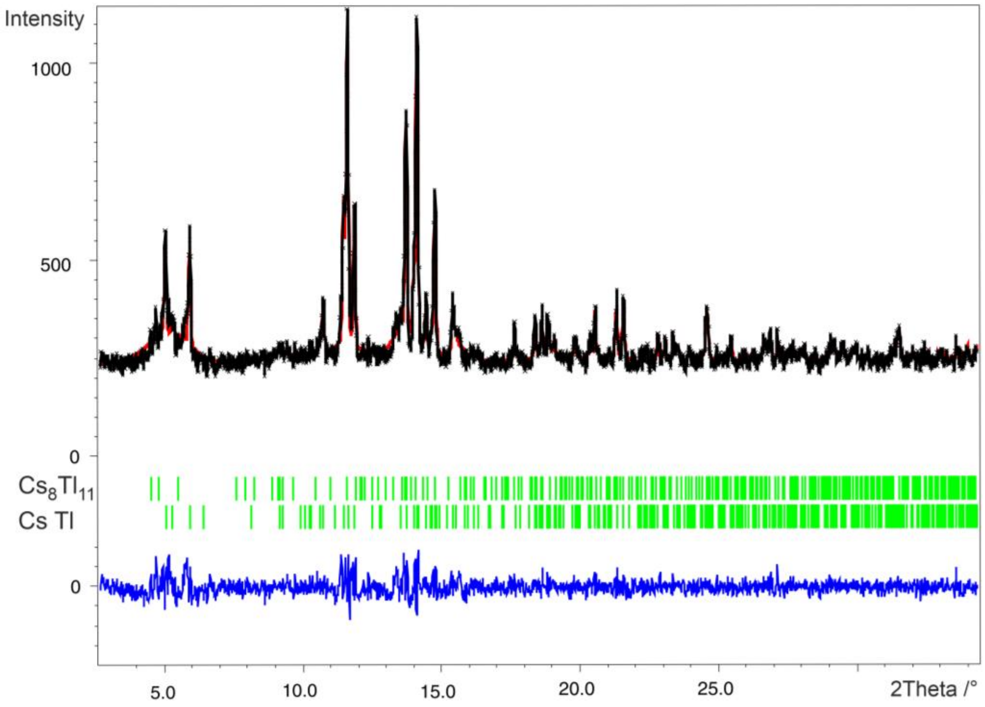

Figure S 12: Measured powder diffraction pattern of the approach CsTl (black). The refinement was carried out with the LeBail algorithm in JANA2006. The vertical bars (green) underneath the powder pattern show the calculated reflection positions. The curve at the bottom (blue) represents the difference plot. GOF=0.99, R_p=4.69, R_{wp}=6.06, x-axis: 2Theta in °, y-axis: Intensity.

2.13 Powder Diffraction Pattern of the Sample Cs_5RbTl_6

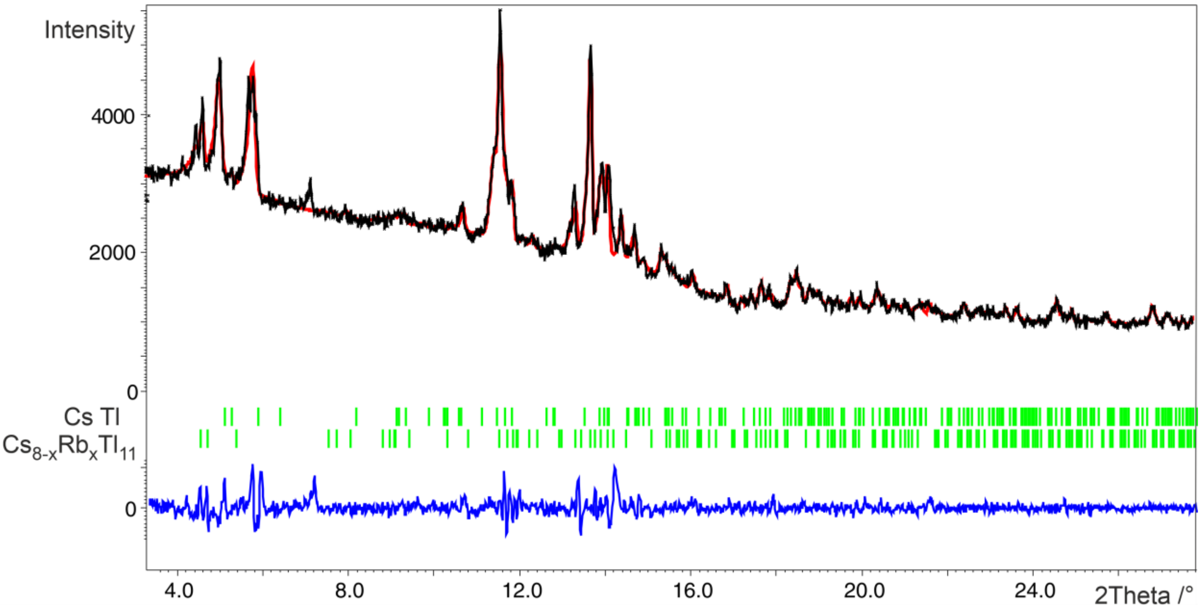

Figure S 13: Measured powder diffraction pattern of the approach Cs_5RbTl_6 (black). The refinement was carried out with the LeBail algorithm in *JANA2006*. The vertical bars (green) underneath the powder pattern show the calculated reflection positions. The curve at the bottom (blue) represents the difference plot. GOF=1.19, R_p=1.87, R_{wp}=2.66, x-axis: 2Theta in °, y-axis: Intensity.

2.14 Powder Diffraction Pattern of the Sample Cs_2RbTl_3 (Temp. 6)

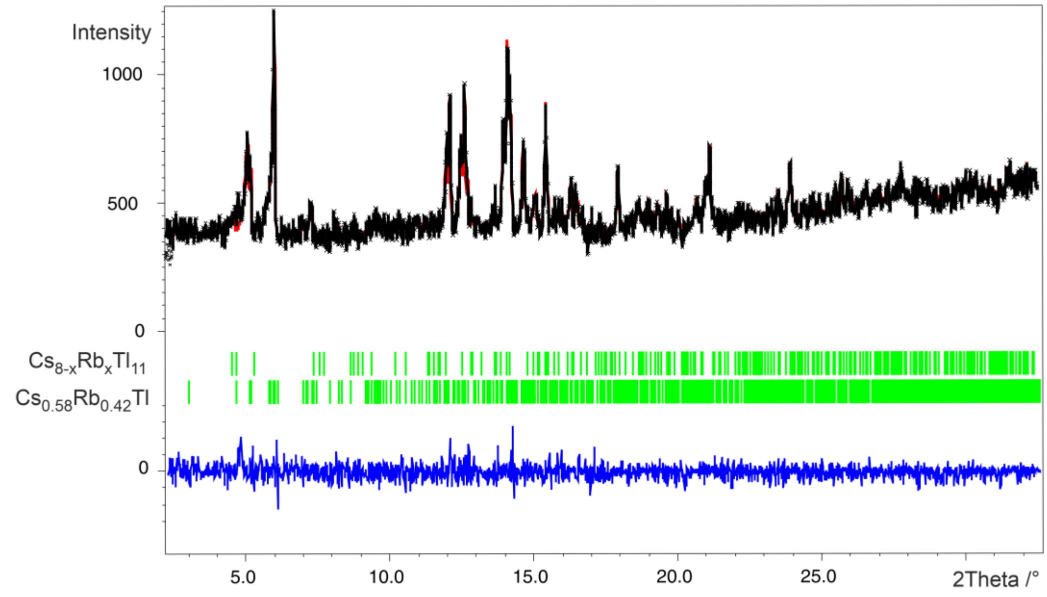

Figure S 14: Measured powder diffraction pattern of the approach Cs_2RbTl_3 (black). The refinement was carried out with the LeBail algorithm in *JANA2006*. The vertical bars (green) underneath the powder pattern show the calculated reflection positions. The curve at the bottom (blue) represents the difference plot. GOF=0.77, R_p=2.06, R_{wp}=2.85, x-axis: 2Theta in °, y-axis: Intensity.

2.15 Powder Diffraction Pattern of the Sample $CsRbTl_2$

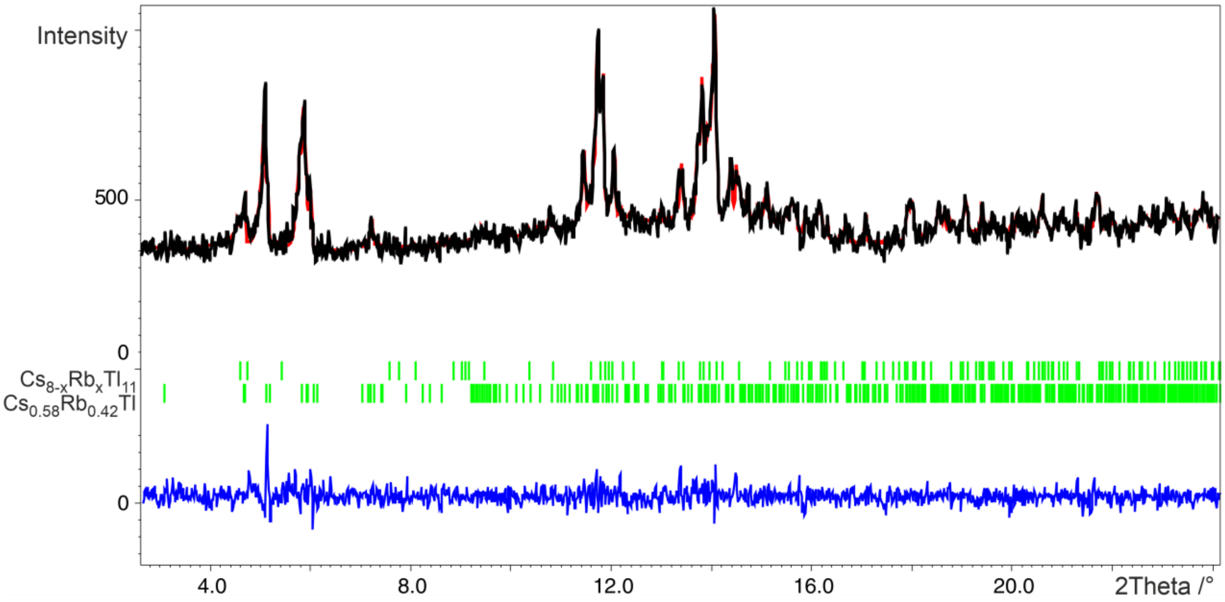

Figure S 15: Measured powder diffraction pattern of the approach $CsRbTl_2$ (black). The refinement was carried out with the LeBail algorithm in *JANA2006*. The vertical bars (green) underneath the powder pattern show the calculated reflection positions. The curve at the bottom (blue) represents the difference plot. GOF=0.73, R_p=2.11, R_{wp}=3.04, x-axis: 2Theta in °, y-axis: Intensity.

2.16 Powder Diffraction Pattern of the Sample $Cs_6Rb_4Tl_{10}$

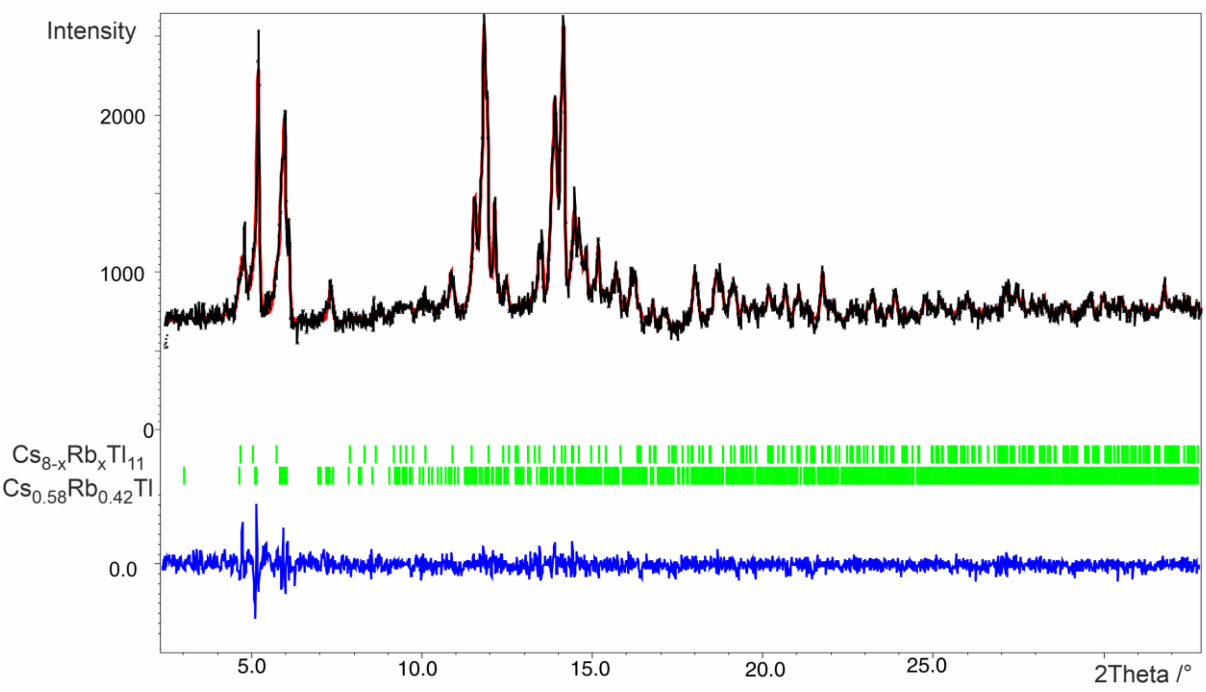

Figure S 16: Measured powder diffraction pattern of the approach $Cs_6Rb_4Tl_{10}$ (black). The refinement was carried out with the LeBail algorithm in *JANA2006*. The vertical bars (green) underneath the powder pattern show the calculated reflection positions. The curve at the bottom (blue) represents the difference plot. GOF=0.84, R_p=1.82, R_{wp}=2.48, x-axis: 2Theta in °, y-axis: Intensity.

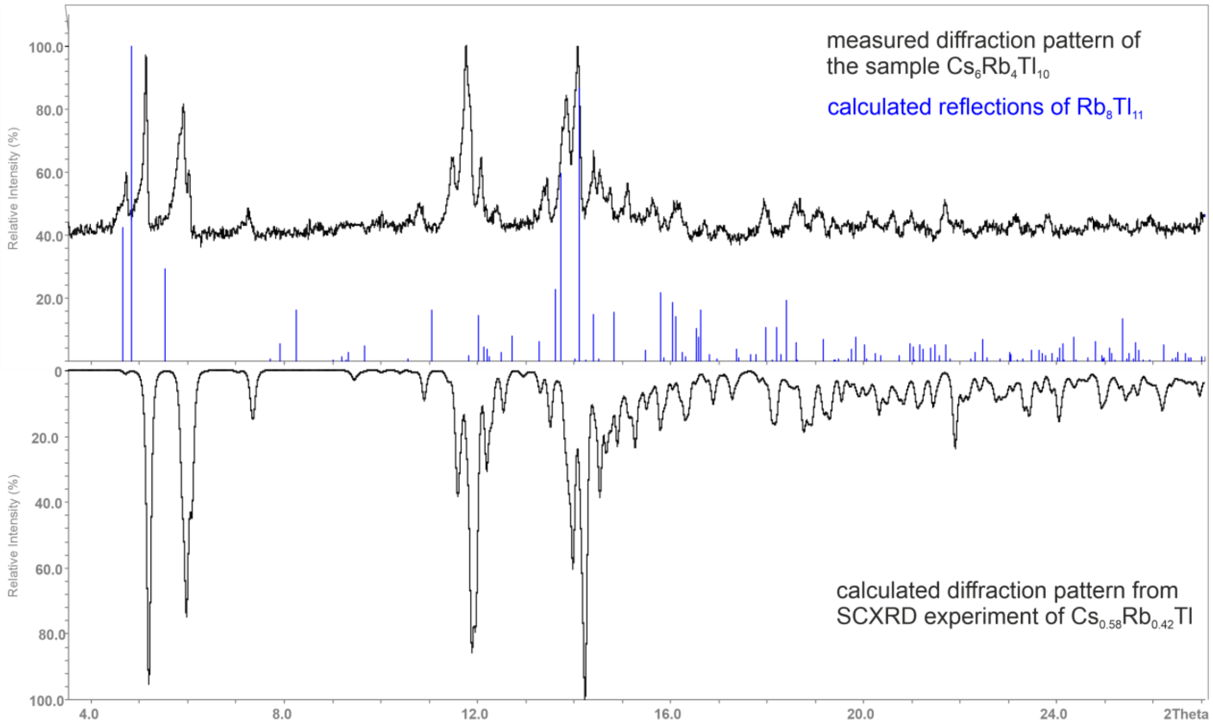

Figure S 17: Measured powder diffraction pattern of the sample $Cs_6Rb_4Tl_{10}$ (black, up) with the calculated reflection of Rb_8Tl_{11} (blue, up) as representative for the $Cs_{8-x}Rb_xTl_{11}$ side product in the sample and the calculated diffraction pattern from the SCXRD experiment of $Cs_{0.58}Rb_{0.42}Tl$ (black, down). , x-axis: 2Theta in °, y-axis: relative intensity./%

2.17 Powder Diffraction Pattern of the Sample $Cs_{0.58}Rb_{0.42}Tl$

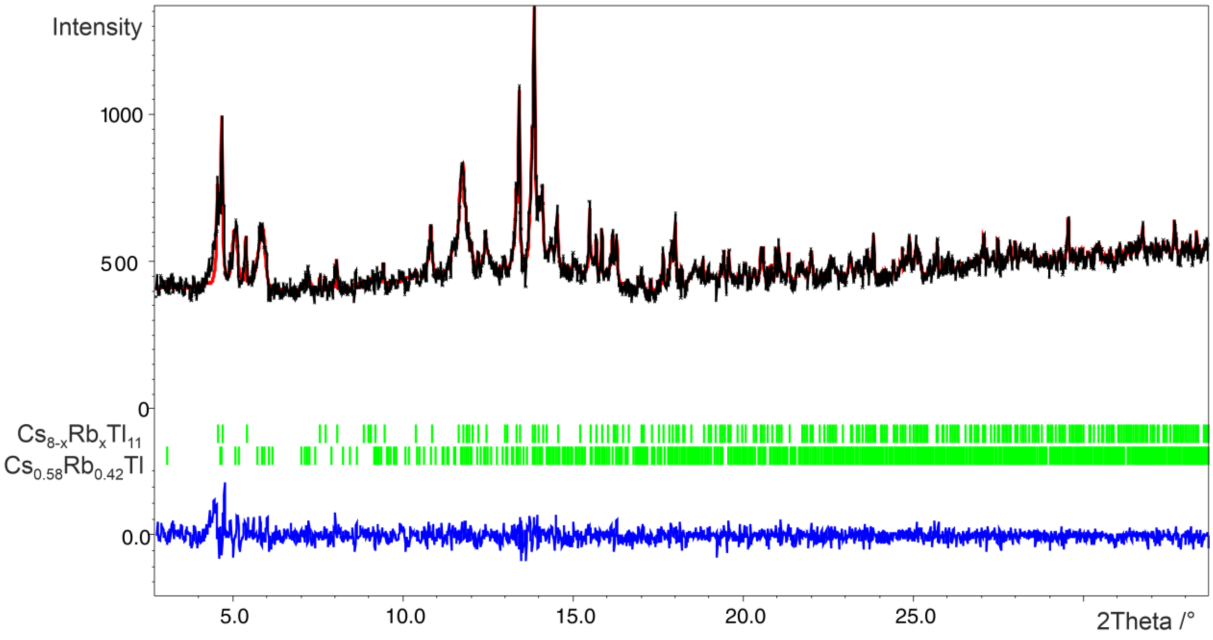

Figure S 18: Measured powder diffraction pattern of the approach $Cs_{0.58}Rb_{0.42}Tl$ (black). The refinement was carried out with the LeBail algorithm in *JANA2006*. The vertical bars (green) underneath the powder pattern show the calculated reflection positions. The curve at the bottom (blue) represents the difference plot. GOF=0.86, R_p=2.46, R_{wp}=3.41, x-axis: 2Theta in °, y-axis: Intensity.

2.18 Powder Diffraction Pattern of the Sample $CsRb_2Tl_3$

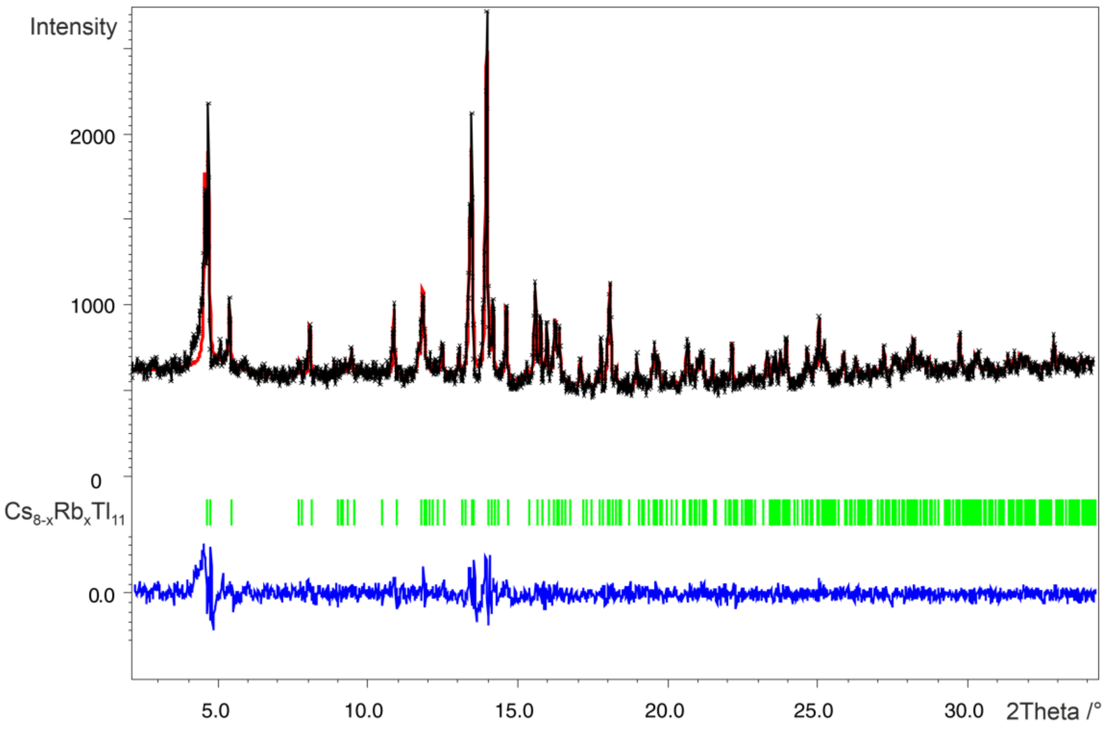

Figure S 19: Measured powder diffraction pattern of the approach $CsRb_2Tl_3$ (black). The refinement was carried out with the LeBail algorithm in *JANA2006*. The vertical bars (green) underneath the powder pattern show the calculated reflection positions. The curve at the bottom (blue) represents the difference plot. GOF=1.09, R_p=2.65, R_{wp}=3.71, x-axis: 2Theta in °, y-axis: Intensity.

2.19 Powder Diffraction Pattern of the Sample $CsRb_5Tl_6$ (Temp. 6)

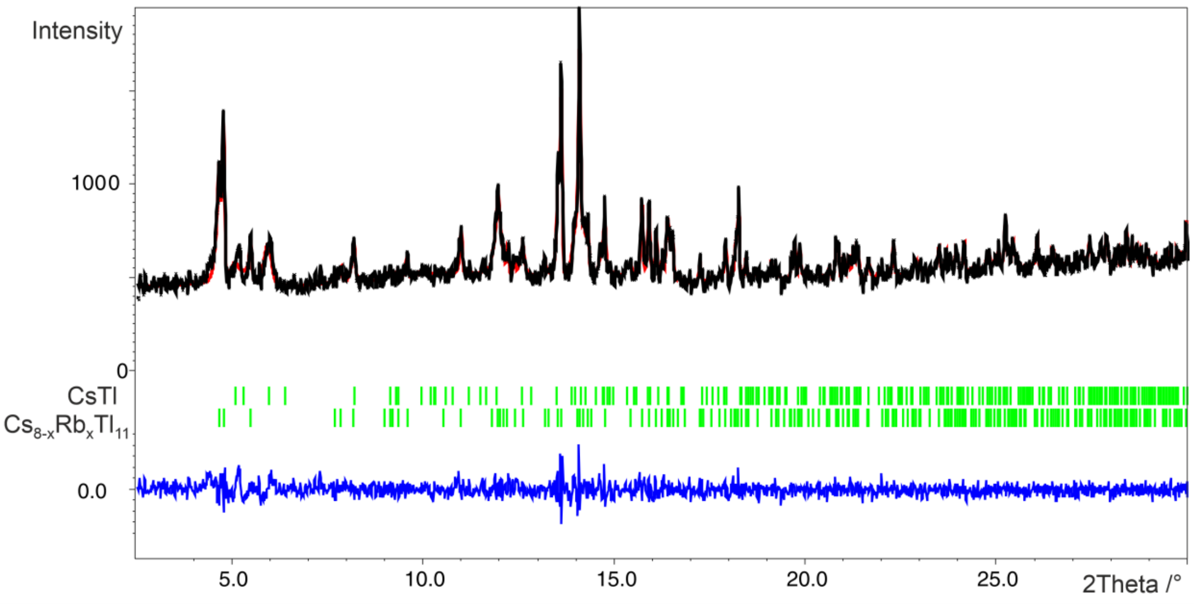

Figure S 20: Measured powder diffraction pattern of the approach $CsRb_5Tl_6$ (black). The refinement was carried out with the LeBail algorithm in *JANA2006*. The vertical bars (green) underneath the powder pattern show the calculated reflection positions. The curve at the bottom (blue) represents the difference plot. GOF=0.88, R_p=2.35, R_{wp}=3.09, x-axis: 2Theta in °, y-axis: Intensity.

3. SEM/EDS Measurements

3.1 Sample Cs_5RbTl_6

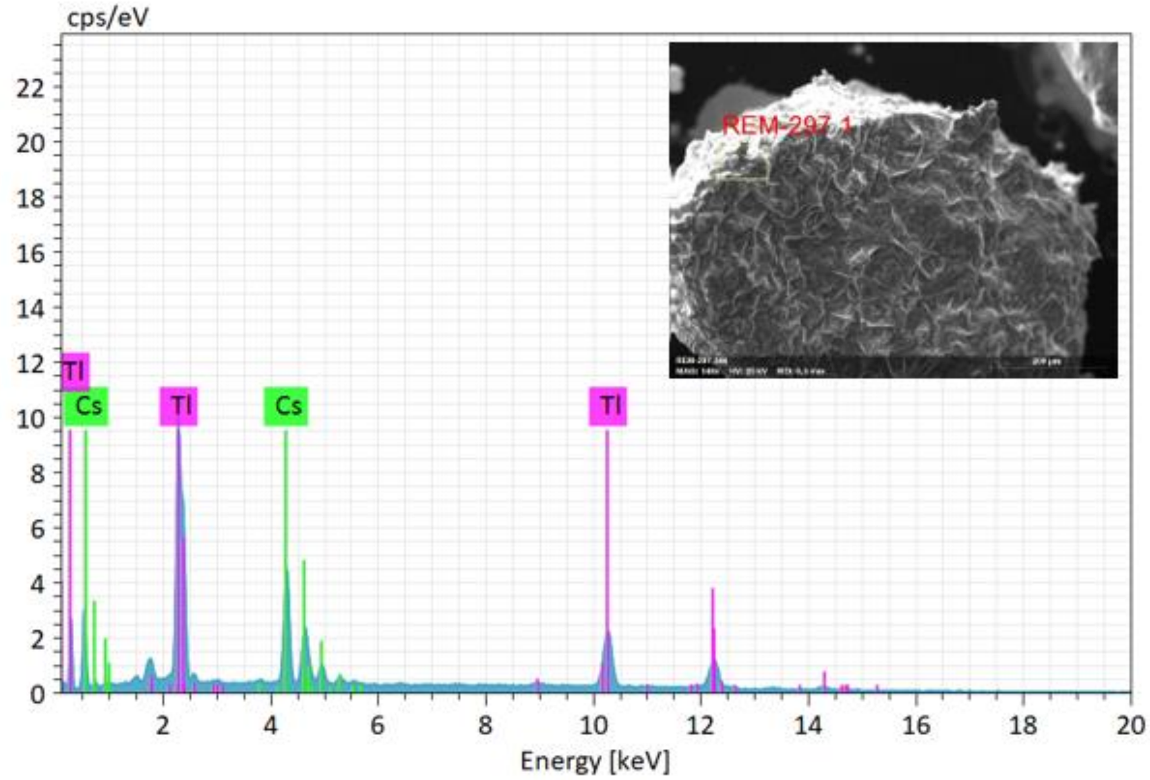

Figure S 21: EDS spectrum and the SEM picture of a crystal from the sample Cs_5RbTl_6.

Table S4: Analysis of the EDS spectrum.

Element	At. No.	Netto	Mass [%]	Mass Norm. [%]	Atom [%]
Tl	81	35106	64.39	62.79	52(5)
Cs	55	51010	38.15	37.21	48(3)

The EDS measurement of a crystal from the sample Cs_5RbTl_6 (see Figure S21 and Table S4) revealed, taking the error into account, that the sample matches the composition CsTl without any hint of rubidium.

A different crystallite from this batch, which was also analyzed with SEM/EDS, showed a liquid surface and due to that a very high amount of cesium (see Figure S22). This example shows, that the alkali metal, especially cesium, from those crystals gets drawn out possibly due to the high vacuum.

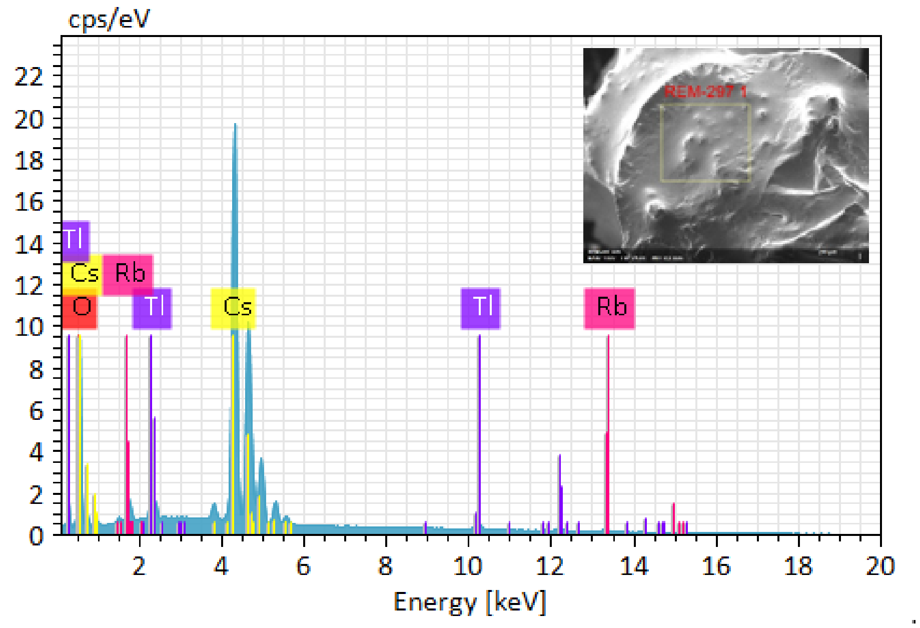

Figure S 22: EDS spectrum and the SEM picture of another crystal from the sample Cs_5RbTl_6.

Table S5: Analysis of the EDS spectrum.

**Element**	**At. No.**	**Netto**	**Mass [%]**	**Mass Norm. [%]**	**Atom [%]**
Tl	81	4531	6.17	6.80	4.4(2)
Rb	37	1251	3.10	3.42	5.3(2)
Cs	55	223724	81.49	89.78	90(2)

3.2 Measurements on the Approach K_5RbTl_6

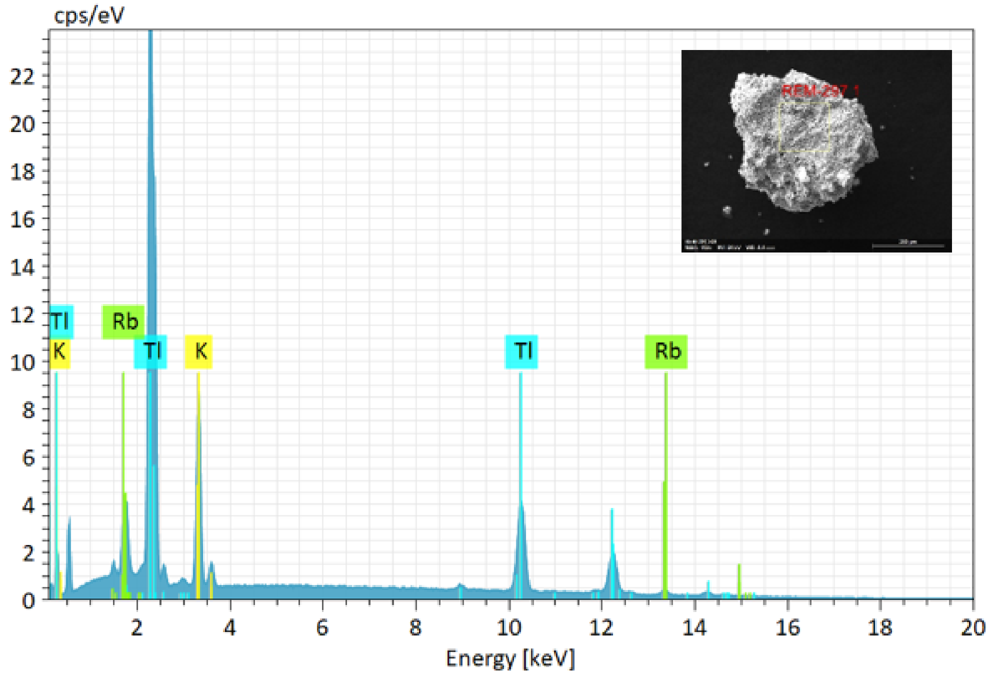

Figure S 23: EDS spectrum and the SEM picture of a crystal from the sample K_5RbTl_6.

Table S6: Analysis of the EDS spectrum.

Element	At. No.	Netto	Mass [%]	Mass Norm. [%]	Atom [%]
Tl	81	58401	74.25	79.68	47(6)
Rb	37	1897	5.02	5.39	7.6(6)
K	19	44644	13.92	14.93	46(1)

The EDS measurement of a crystal from the sample K_5RbTl_6 (see Figure S23 and Table S6) showed, taking the error into account, that the sample matches the composition $K_{0.86}Rb_{0.14}Tl$ quite well (atomic percentage calculated/measured: 50/47(6)% (Tl), 43/46(1)% (K), 7/7.6(6)% (Rb)).

4. Direct Reduction Experiments in Liquid Ammonia

4.1 $TlBF_4$ + 2 Rb + NH_3(l)

Dry ammonia (5 mL) was condensed onto the educts (Rb: 68.4 mg, 0.8 mmol; $TlBF_4$: 116.5 mg, 0.4 mmol), which resulted in a dark blue solution. After two days of storage at 233 K the color changed from dark blue to yellow-green with black sediment. The liquid ammonia was evaporated, and the dried residue was characterized by powder diffraction.

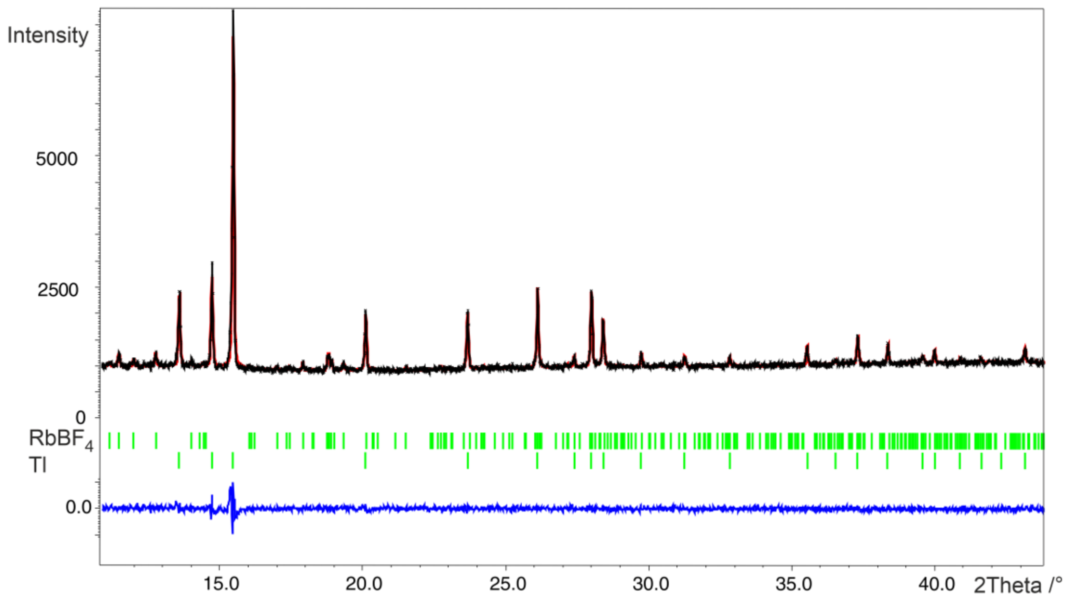

Figure S 24: Measured powder diffraction pattern of the approach $TlBF_4$+2Rb (black). The refinement was carried out with the LeBail algorithm in JANA2006. The vertical bars (green) underneath the powder pattern show the calculated reflection positions. The curve at the bottom (blue) represents the difference plot. GOF=0.97, R_p=2.22, R_{wp}=3.02.

4.2 $TlPF_6$ + 2 Rb + NH_3(l)

Dry ammonia (5mL) was condensed onto the educts (Rb: 68.4 mg, 0.8 mmol; $TlPF_6$: 139.7 mg, 0.4 mmol), which resulted in a dark blue solution. After two days of storage at 233 K the color changed

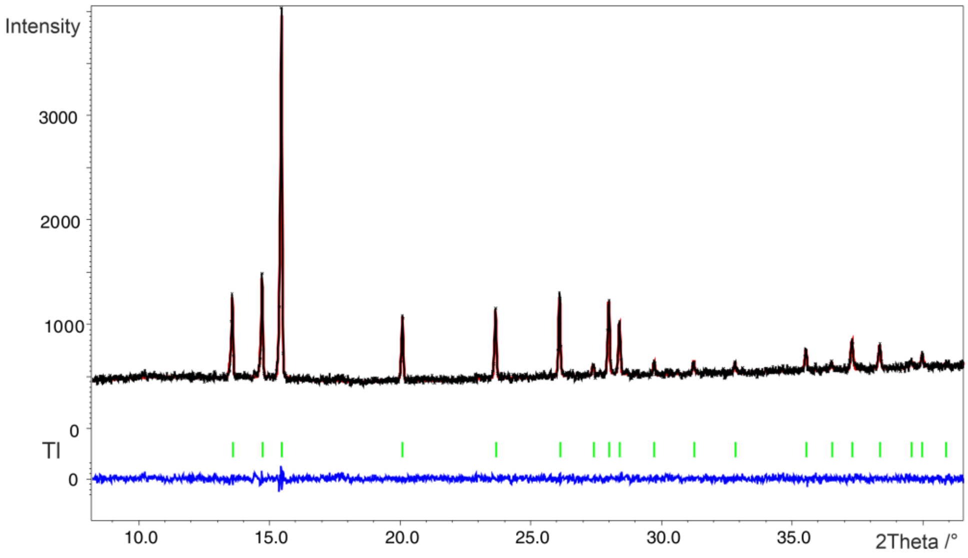

Figure S 25: Measured powder diffraction pattern of the approach $TlPF_6$+2Rb (black). The refinement was carried out with the LeBail algorithm in *JANA2006*. The vertical bars (green) underneath the powder pattern show the calculated reflection positions. The curve at the bottom (blue) represents the difference plot. GOF=0.86, R_p=2.89, R_{wp}=3.67.

from dark blue to yellow with black sediment. The liquid ammonia was evaporated, and the dried residue was characterized by powder diffraction.

4.3 TlBr + 2 Rb + NH_3(l)

Dry ammonia (5 mL) was condensed onto the educts (Rb: 338.7 mg, 0.8 mmol; TlBr: 405.0 mg, 1.982 mmol) in an H-tube, which resulted in a dark blue solution. After five days of storage at 233 K the color changed from dark blue to pale yellow with grey sediment. The residue was washed twice through filtering in the second half of the H-tube, and afterward, ammonia was again condensed on it. Later, the liquid ammonia was evaporated, and the dried residues were tried to be characterized by powder diffraction. Due to the statically charged white sample particles, filling into capillaries was unfortunately hardly possible, and the quantity filled was therefore not sufficient to record a powder diffraction pattern. When ground, the fluffy grey residue became a slivery, shiny plate that was hard and brittle. Single crystal data of small pieces of this showed, that it was elemental thallium.

5. Distances of Compressed $[Tl_6]^{6-}$ Octahedra

Table S7: Distances and degree of distortion of compressed $[Tl_6]^{6-}$ octahedra, which are known so far.

**Compounds**	$d(Tl_{eq} - Tl_{eq})$ /Å	$d(Tl_{eq} - Tl_{ap})$ /Å	$d(Tl_{ap} - Tl_{ap})$ /Å	$d_{ap}/\overline{d_{eq}}$
**KTl***	3.3329(6)-3.4585(6)	3.0471(5)-3.0710(5)	3.7898(9)	1.12
**$K_{0.86}Rb_{0.14}Tl$**	3.3347(7)-3.4566(7)	3.0506(6)-3.0696(6)	3.7934(10)	1.12
**$K_{0.72}Rb_{0.28}Tl$**	3.3281(4)-3.4505(4)	3.0464(3)-3.0636(3)	3.7881(6)	1.12
**$K_{0.54}Rb_{0.46}Tl$**	3.3376(6)-3.4575(6)	3.0522(4)-3.0691(4)	3.7917(8)	1.12
**$K_{0.31}Rb_{0.69}Tl$**	3.3441(4)-3.4635(4)	3.0503(3)-3.0688(3)	3.7769(5)	1.11
**CsTl***	3.3932(8)-3.4130(7)	3.0216(5)-3.0772(5)	3.7477(10)	1.10
**$Cs_{0.58}Rb_{0.42}Tl$**	3.3767(4)-3.4262(4)	3.0219(4)-3.1044(4)	3.7680(4)	1.11
**$Cs_{0.82}Rb_{0.18}Tl$**	3.377(1)-3.435(1)	3.015(1)-3.107(1)	3.750(1)	1.10
	3.35(2)-3.46(3)	2.91(3)-3.21(3)	3.85(3)	1.13
	3.3752(9)-3.4088(9)	3.045(1)-3.0672(9)	3.785(1)	1.12
**$Cs_{7.29}K_{5.71}Tl_{13}$** [5]	3.3418(3)-3.3687(3)	3.0143(3)-3.1203(3)	3.8819(4)	1.16
**$Rb_{10}Tl_6O_2$** [6]	3.415(2)-3.654(2)	3.014(1)-3.090(1)	3.574(2)	1.01
**$K_{10}Tl_6O_2$** [6]	3.390(1)-3.704(1)	3.010(1)-3.094(1)	3.578(1)	1.01
**$Cs_{10}Tl_6SiO_4$** [7]	3.388(2)-3.394(2)	3.001(2)-3.116(2)	3.772(7)	1.11
	3.407(2)-3.414(2)	3.035(2)-3.117(2)	3.800(7)	1.11
**$Cs_{10}Tl_6SnO_3$** [7]	3.394(1)-3.474(1)	3.033(1)-3.105(1)	3.371(1)	0.98

* Redetermination at 123 K.

6. CsTl

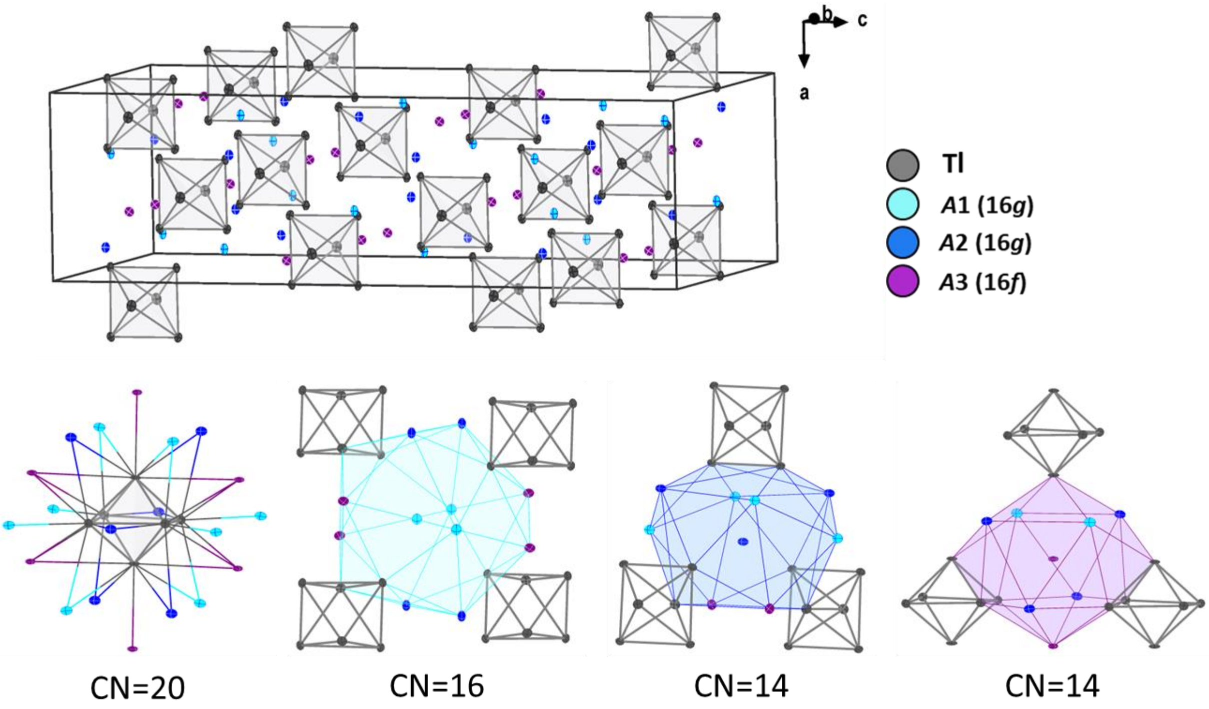

Figure S 26: Unit cell, cluster coordination, and alkali metal coordination of the structure type CsTl.

7. Trend of the Solid Solution $K_{1-x}Rb_xTl$ ($x \leq 0.69$)

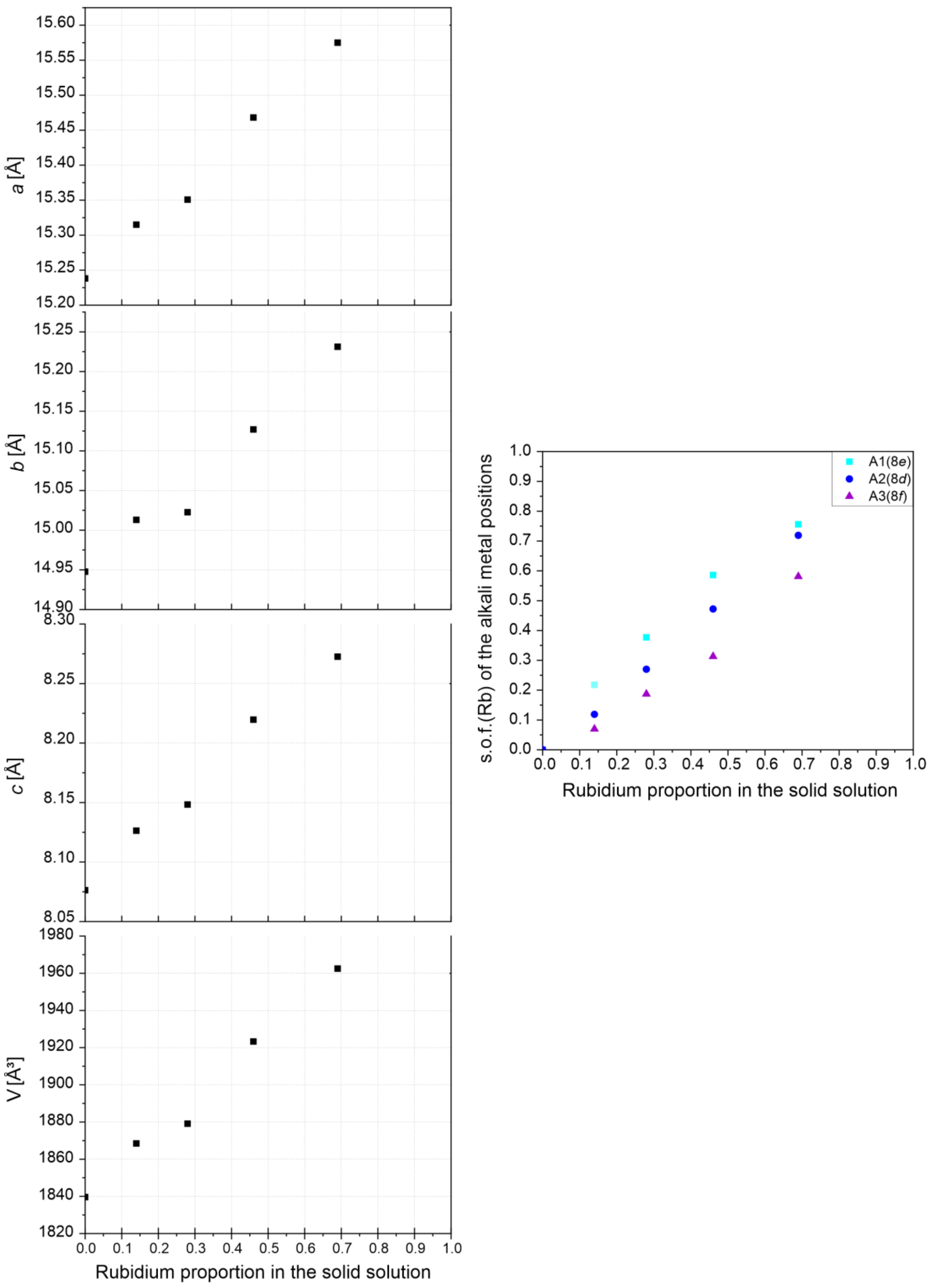

Figure S 27: Trends of the cell parameters, volume, and site occupancy factors in the solid solution $K_{1-x}Rb_xTl$ ($x \leq 0.69$).

8. $Cs_{0.58}Rb_{0.42}Tl$

8.1 Atomic Coordinates

Table S8: Fractional atomic coordinates and equivalent isotropic displacement parameters for $Cs_{0.58}Rb_{0.42}Tl$. U_{eq} is defined as 1/3 of the trace of the orthogonalised U_{IJ} tensor.

**Atom (Wyckoff)**	***x***	***y***	***z***	***U*(eq) /Å²**
Tl1 (8*f*)	0.10267(2)	0.02582(2)	0.15397(1)	0.01769(5)
Tl2 (8*f*)	0.18022(2)	0.06755(3)	0.06208(1)	0.01964(6)
Tl3 (8*f*)	0.18458(2)	0.45897(3)	0.31060(1)	0.01918(6)
Tl4 (8*f*)	0.28578(2)	0.30556(2)	0.39843(1)	0.01771(5)
Tl5 (8*f*)	0.29031(2)	0.21664(2)	0.15176(1)	0.01854(5)
Tl6 (8*f*)	0.39170(2)	0.00107(2)	0.09453(1)	0.01835(5)
Cs1 (8*f*)	0.07027(5)	0.33696(6)	0.18727(3)	0.0206(2)
Cs2 (8*f*)	0.09386(4)	0.18381(5)	0.45736(2)	0.0201(2)
Cs3 (8*f*)	0.15120(4)	0.05539(5)	0.29994(2)	0.021.9(2)
Cs4 (8*f*)	0.15131(4)	0.45474(5)	0.0494.4(2)	0.0213(2)
Cs5 (8*f*)	0.41523(4)	0.20930(5)	0.29639(2)	0.0221(2)
Cs6 (8*f*)	0.43876(4)	0.31564(5)	0.06446(2)	0.0196(2)
Rb1 (8*f*)	0.07027(5)	0.33696(6)	0.18727(3)	0.0207(2)
Rb2 (8*f*)	0.09386(4)	0.18381(5)	0.45736(2)	0.0202(2)
Rb3 (8*f*)	0.15120(4)	0.05539(5)	0.29994(2)	0.0219(2)
Rb4 (8*f*)	0.15131(4)	0.045474(5)	0.04944(2)	0.0214(2)
Rb5 (8*f*)	0.41523(4)	0.20930(5)	0.29639(2)	0.0221(2)
Rb6 (8*f*)	0.43876(4)	0.31564(5)	0.06446(2)	0.0196(2)

8.2 Displacement Parameter

Table S9: Anisotropic Displacement Parameters for $Cs_{0.58}K_{0.42}Tl$. The Anisotropic displacement factor exponent takes the form: $-2\pi^2[h^2a^{*2}U_{11}+2hka^*b^*U_{12}+\ldots]$.

**Atom**	U_{11}	U_{22}	U_{33}	U_{12}	U_{13}	U_{23}
Tl1	0.0178(1)	0.0157(1)	0.0211(1)	0.00041(8)	0.00775(9)	-0.00174(9)
Tl2	0.0211(1)	0.0218(1)	0.0137(1)	0.00246(9)	-0.00023(9)	0.00258(9)
Tl3	0.0210(1)	0.0219(1)	0.0129(1)	-0.00268(9)	0.00093(9)	-0.00233(9)
Tl4	0.0212(1)	0.01318(9)	0.0203(1)	0.00110(8)	0.008.03(9)	0.00240(8)
Tl5	0.0214(1)	0.01397(9)	0.0210(1)	-0.00196(8)	0.00671(9)	-0.00349(9)
Tl6	0.0192(1)	0.0170(1)	0.0210(1)	0.00220(8)	0.00909(9)	0.00177(9)
Cs1	0.0235(3)	0.0182(3)	0.0196(3)	0.0036(2)	0.0038(2)	-0.0027(2)
Cs2	0.0218(2)	0.0190(2)	0.0202(3)	-0.0025(2)	0.0059(2)	0.0029(2)
Cs3	0.0218(2)	0.0227(2)	0.0202(3)	0.0009(2)	0.0029(2)	0.0009(2)
Cs4	0.0225(2)	0.0224(2)	0.0185(3)	-0.0001(2)	0.0036(2)	0.0009(2)
Cs5	0.0260(2)	0.0215(2)	0.0202(3)	-0.0018(2)	0.0084(2)	-0.0051(2)
Cs6	0.0224(3)	0.0190(3)	0.0173(3)	-0.0012(2)	0.0045(2)	0.0027(2)
Rb1	0.0235(3)	0.0182(3)	0.0196(3)	0.0036(2)	0.0038(2)	-0.0027(2)
Rb2	0.0218(2)	0.0190(2)	0.0202(3)	-0.0025(2)	0.0059(1)	0.0029(2)
Rb3	0.0218(2)	0.0227(2)	0.0202(3)	0.0009(2)	0.0029(2)	0.0009(2)
Rb4	0.0225(2)	0.0224(2)	0.0185(3)	-0.0001(2)	0.0036(2)	0.0009(2)
Rb5	0.0260(2)	0.0215(2)	0.0202(3)	-0.0018(2)	0.0084(2)	-0.0051(2)
Rb6	0.0224(3)	0.0190(3)	0.0173(3)	-0.0012(2)	0.0045(2)	0.0027(2)

8.3 Distances

Table S10: Selected atomic distances of the first coordination sphere in $Cs_{0.58}K_{0.48}Tl$.

Tl1 (8*f*)		Tl2 (8*f*)		Tl3 (8*f*)	
Tl2	3.0350(4)	Tl3	3.7680(4)	Tl4	3.0251(4)
Tl3	3.0448(4)	Tl4	3.1045(4)	Tl5	3.0366(4)
Tl4	3.4238(4)	Tl5	3.0712(6)	Tl6	3.1014(8)
Tl5	3.4262(4)	Tl6	3.0219(4)	Cs1/Rb1	3.6546(9)<br>3.8927(8)
Cs1/Rb1	3.6351(7)	Cs2/Rb2	3.9902(7)<br>4.0268(6)	Cs3/Rb3	4.4022(8)<br>4.5116(6)
Cs2/Rb2	4.0219(6)	Cs4/Rb4	4.3279(6)<br>4.3438(8)	Cs5/Rb5	4.0519(7)<br>4.3926(7)<br>4.6813(6)
Cs3/Rb3	3.9263(7)<br>4.1307(7)	Cs6/Rb6	3.7218(7)<br>4.4502(6)<br>4.5916(6)		
Cs5/Rb5	3.8046(6)				
Cs6/Rb6	3.7688(7)				
**Tl4 (8*f*)**		**Tl5 (8*f*)**		**Tl6 (8*f*)**	
Tl6	3.3767(4)	Tl6	3.3799(4)	Cs1/Rb1	3.6304(7)
Cs2/Rb2	3.7573(7)<br>3.9293(7)	Cs1/Rb1	3.7528(9)	Cs2/Rb2	3.8286(6)
Cs3/Rb3	3.0308(6)	Cs3/Rb3	4.0120(6)	Cs4/Rb4	3.8958(6)<br>4.2132(7)
Cs4/Rb4	3.1759(6)	Cs4/Rb4	4.0205(6)	Cs5/Rb5	4.2369(6)
Cs5/Rb5	3.8706(7)	Cs5/Rb5	3.9520(7)<br>4.0950(6)	Cs6/Rb6	3.6912(7)
Cs6/Rb6	3.8129(7)	Cs6/Rb6	3.7356(7)		
**Cs1/Rb1 (8*f*)**		**Cs2/Rb2 (8*f*)**		**Cs3/Rb3 (8*f*)**	
Cs1/Rb1	4.397(2)	Cs2/Rb2	4.736(1)	Cs3/Rb3	4.5176(11)
Cs2/Rb2	4.4376(9)	Cs3/Rb3	4.8192(8)	Cs4/Rb4	4.5513(7)
Cs3/Rb3	4.3565(9)<br>4.4139(9)<br>4.5919(9)	Cs4/Rb4	4.4734(8)<br>4.5785(8)<br>4.7095(8)	Cs5/Rb5	4.1529(8)<br>4.6344(8)
Cs4/Rb4	4.426(1)	Cs6/Rb6	4.0930(9)<br>4.1414(9)	Cs6/Rb6	5.0002(9)
Cs5/Rb5	4.1590(9)				
**Cs4/Rb4 (8*f*)**		**Cs5/Rb5 (8*f*)**		**Cs6/Rb6 (8*f*)**	
Cs4/Rb4	4.6206(10)	Cs5/Rb5	3.9227(12)		
Cs6/Rb6	4.3013(9)<br>4.3053(9)	Cs6/Rb6	4.0653(7)		

8.4 DSC Measurements

DSC analysis was carried out on the sample $Cs_6Rb_4Tl_{10}$ under a flow of nitrogen (sample purge flow: N_2 40 mL/min). The four heating/cooling cycles were performed from 293.15 K to 593.15 K at a 10 K/min rate. This cooling rate simulates the quenching applied to the sample itself to obtain the desired compound $Cs_{0.58}Rb_{0.42}Tl$ (quenching from 773 K with water or liquid nitrogen (see Chapter 2, SI)). Th sample was precalcinated at 300 °C due to inhomogeneity. Effects upon this treatment cannot be excluded.

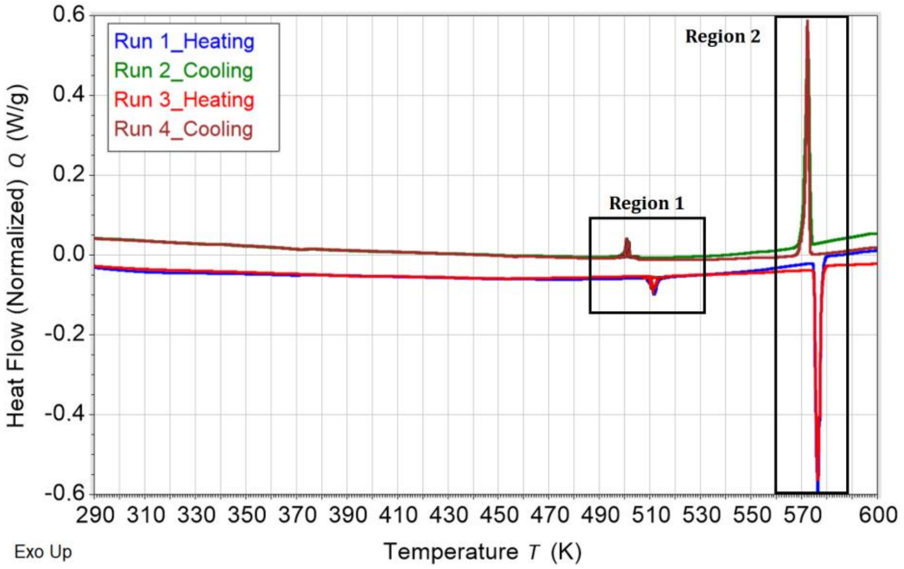

Figure S 28: Two cycles of a DSC measurement of the sample $Cs_6Rb_4Tl_{10}$ in a closed system.

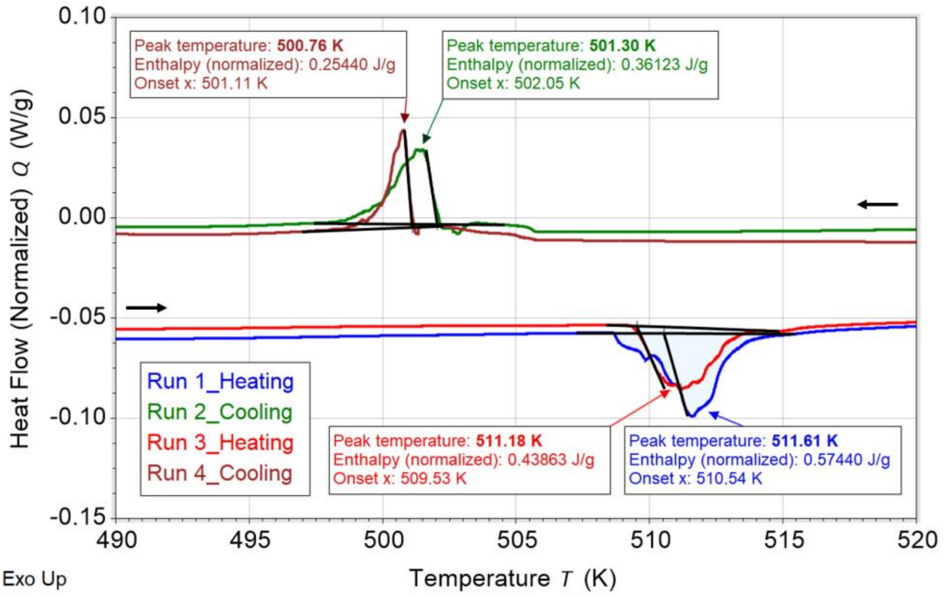

Figure S 29: Zoom in region 1 (490-520 K) of the two cycle DSC measurement of the sample $Cs_6Rb_4Tl_{10}$.

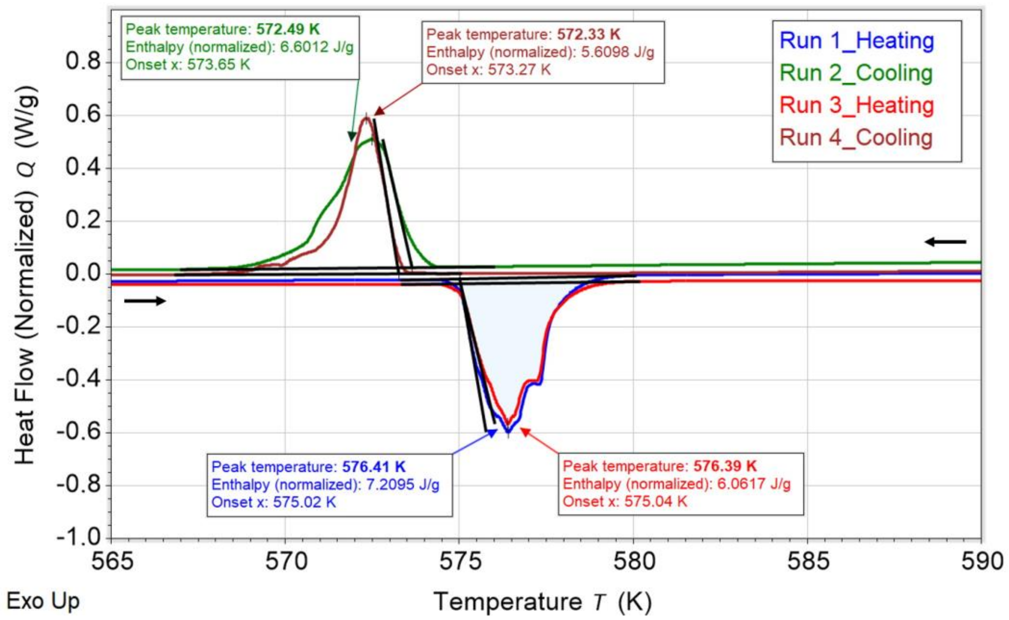

Figure S 30: Zoom in the region 2 (565-590 K) of the DSC measurement of the sample $Cs_6Rb_4Tl_{10}$.

8.5 Temperature Dependent PXRD

The temperature program for the temperature-dependent PXRD measurements had the following steps (N1-20): 298 K, 373 K, 423 K, 473 K, 493 K, 498 K, 503 K, 508 K, 513 K, 518 K, 523 K, 518 K, 513 K, 508 K, 503 K, 498 K, 493 K, 423 K, 373 K, 298 K.

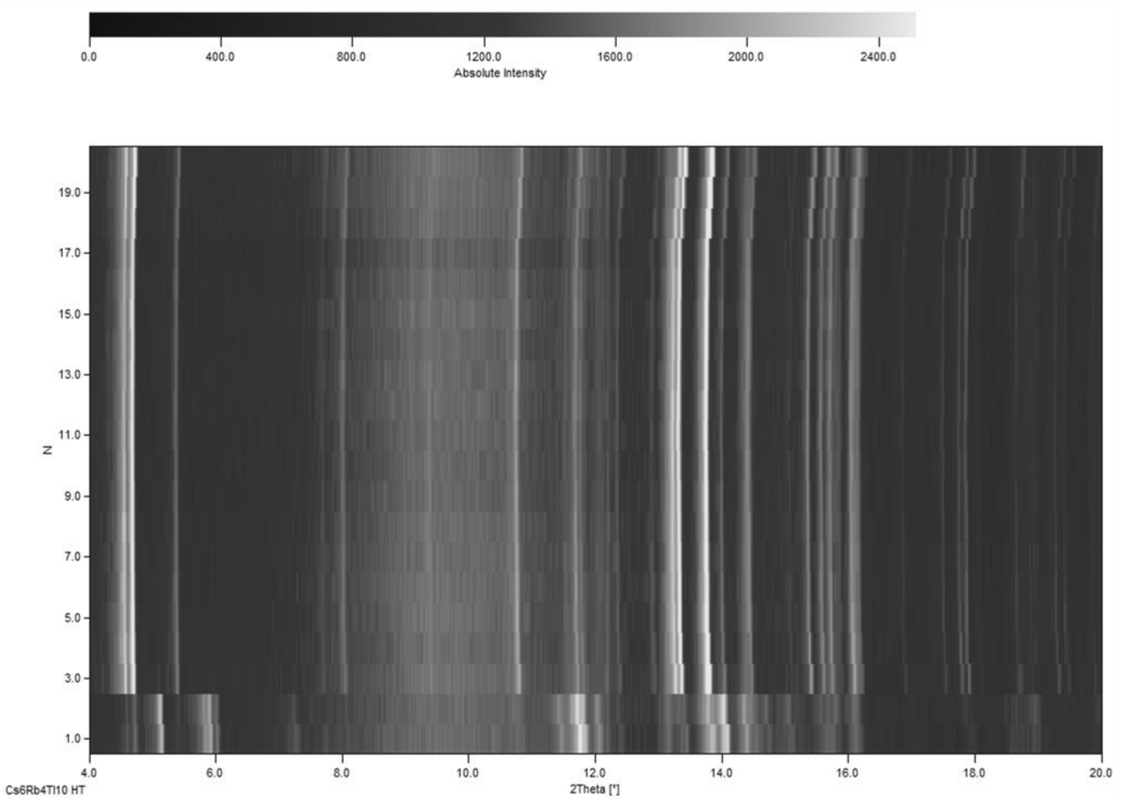

Figure S 31: Temperature dependent PXRD measurements of the sample $Cs_6Rb_4Tl_{10}$. x-axis: 2Theta [°], y-axis: number of cycle.

Due to the temperature holding time for measuring the powder pattern (2 h), this temperature-dependent analysis is comparable with a slow cooling rate and annealing of the product.

It can stated in this case, that at 423 K the desired product is no more present, but only the $Cs_{8-x}Rb_xTl_{11}$ remains during the rest of the heating and cooling process.

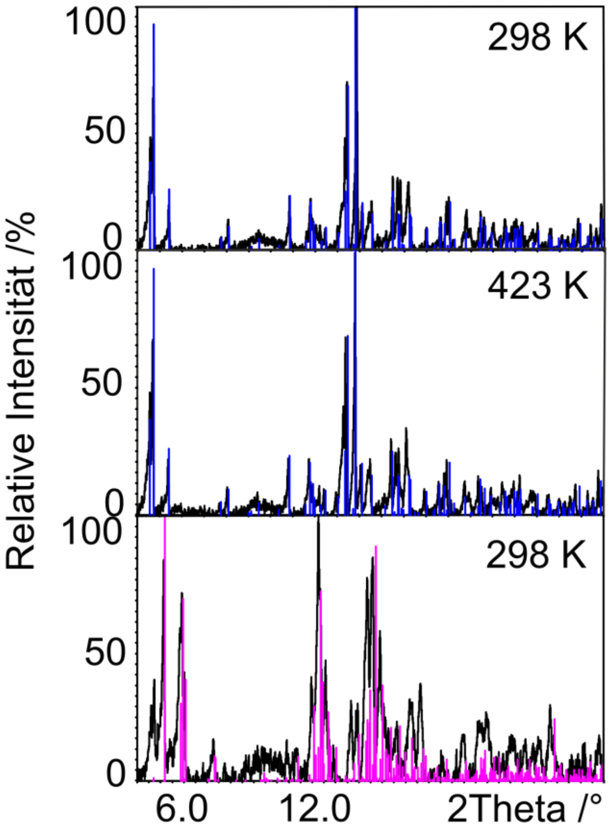

Figure S 32: Relevant PXRD patterns from the temperature-dependent measurements of the sample $Cs_5Rb_4Tl_{10}$. Starting at the bottom is the starting sample at room temperature with the calculated reflection pattern of $Cs_{0.58}Rb_{0.42}Tl$ (pink). In the middle is the pattern at 423 K shown with the calculated reflections of Cs_8Tl_{11} (blue). The pattern at the top shows the result after cooling back down to room temperature with the calculated reflections of Cs_8Tl_{11} (blue).

9. $Cs_{0.82}Rb_{0.18}Tl$

9.1 Structure Description

The asymmetric unit consists of nine crystallographic different thallium and alkali metal positions on a general Wyckoff position 8*f*. The thallium atoms form two discrete compressed [Tl_6] octahedra, from which at least one shows a distortion, which occurs in a rotation of the cluster (see Figure S33).

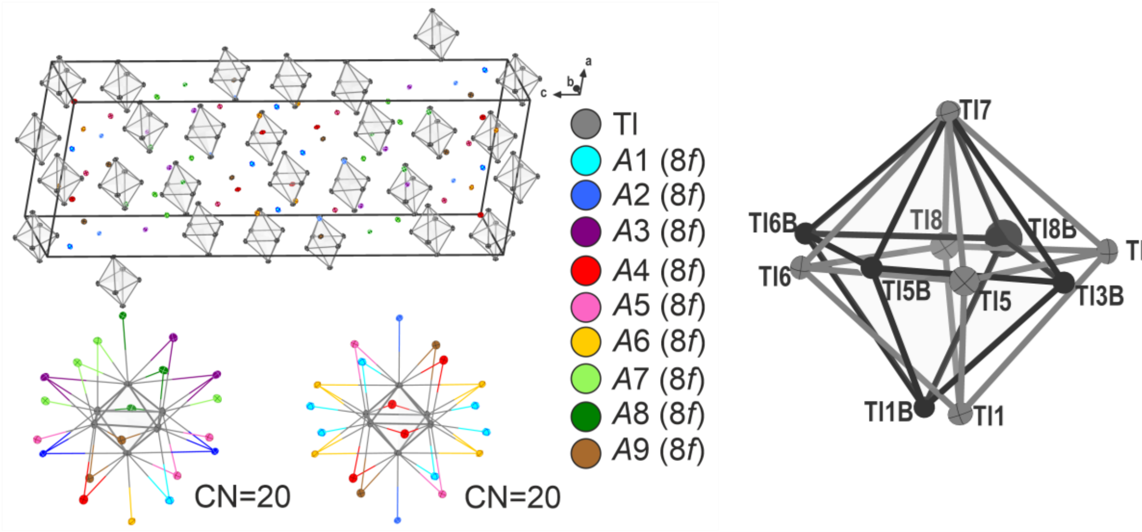

Figure S 33: Unit cell, cluster coordinations, and cluster disorder in $Cs_{0.82}Rb_{0.18}Tl$.

The [Tl_6] octahedra are surrounded by 20 alkali metal atoms (d(Tl-*A*≤4.7 Å)), like in the other structure types $K_{1-x}Rb_xTl$ (x≤0.69) and $Cs_{1-x}Rb_xTl$ (x=0, 0.42). Six of the alkali metal atoms are exo-coordinating to each vertex of the octahedron. Additionally, there are four face-capping and ten edge-capping alkali metal atoms.

All nine crystallographically different alkali metal positions are mixed occupied by cesium and rubidium. The coordination numbers vary from 13 to 14 and 16. They can be differentiated first of all in coordinations with four (*A*1, *A*5, *A*6) and three $[Tl_6]^{6-}$ octahedra (*A*2, *A*3, *A*4, *A*7, *A*8, *A*9) (see Figure S34).

In general, the coordination polyhedra are similar to those found in binary KTl and CsTl (Figure S23, and Figure 2 in main text). The alkali metal positions *A*1, *A*5, and *A*7 are coordinated similarly to Cs1 (Wyckoff 16*g*) in CsTl. The ternary materials when mixing rubidium and cesium combine structural features of KTl and CsTl. While the coordination sphere of *A*4 and *A*9 are similar to that of K2 (Wyckcoff 8d) in KTl and Cs2 (Wyckoff 16*g*) in CsTl, positions *A*2 and *A*3 are surrounded like Cs3 (Wyckoff 8*e*) in CsTl. In contrast to $Cs_{0.58}Rb_{0.42}Tl$ there is the first coordination sphere of *A*8, which is similar to the surrounding of K3 (Wyckoff 8f) in KTl. In addition, *A*3 shows also an expanded coordination sphere in comparison to Cs3. Another difference is the coordination of the third [Tl_6] cluster, on which *A*3 is edge coordinating instead of vertex coordinating.

The occupation trend from low to high rubidium ratio is in accordance with the different coordination numbers and follows this order: *A*1<*A*5<*A*7<*A*9, *A*3<*A*4<*A*6<*A*2<*A*8.

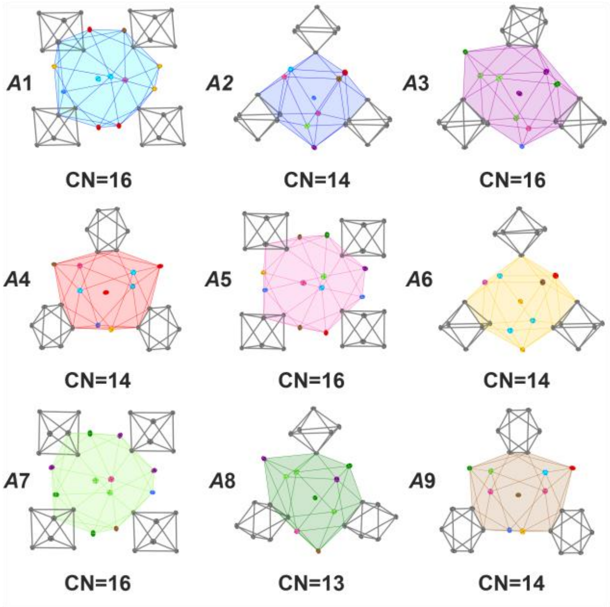

Figure S 34: The first coordination spheres of the nine crystallographically different alkali metal positions in $Cs_{0.82}Rb_{0.18}Tl$.

9.2 Atomic Coordinates

Table S11: Fractional atomic coordinates and equivalent isotropic displacement parameters for $Cs_{0.58}Rb_{0.42}Tl$. U_{eq} is defined as 1/3 of the trace of the orthogonalised U_{IJ} tensor.

**Atom (Wyckoff)**	***x***	***y***	***z***	***U*(eq)**
Tl1 (8*f*)	0.16882(5)	0.46189(8)	0.31329(2)	0.01699(14)
Tl2 (8*f*)	0.52157(5)	0.05543(6)	0.45767(2)	0.01600(14)
Tl3 (8*f*)	0.35694(5)	0.27204(6)	0.31548(2)	0.01746(14)
Tl4 (8*f*)	0.33656(5)	-0.01606(5)	0.48177(2)	0.01446(13)
Tl5 (8*f*)	0.30519(5)	0.42064(6)	0.37409(2)	0.01878(15)
Tl6 (8*f*)	0.31342(5)	0.68102(6)	0.34867(2)	0.01594(14)
Tl7 (8*f*)	0.49392(5)	0.48615(6)	0.35408(2)	0.01700(14)
Tl8 (8*f*)	0.35645(5)	0.52874(6)	0.29095(2)	0.01731(15)
Tl9 (8*f*)	0.47912(5)	0.20307(5)	0.51696(2)	0.01446(13)
Cs1 (8*f*)	0.55104(9)	0.44156(10)	0.45045(3)	0.0208(4)
Cs2 (8*f*)	0.56351(9)	0.17060(10)	0.37361(3)	0.0174(4)
Cs3 (8*f*)	0.11571(9)	0.77817(11)	0.28071(3)	0.0221(4)
Cs4 (8*f*)	0.71341(9)	0.32778(10)	0.55378(3)	0.0199(4)
Cs5 (8*f*)	0.28619(9)	0.03175(10)	0.38424(3)	0.0204(4)
Cs6 (8*f*)	0.27433(9)	0.30508(10)	0.45680(3)	0.0181(4)
Cs7 (8*f*)	0.11739(9)	0.43168(10)	0.21677(3)	0.0204(4)
Cs8 (8*f*)	0.11372(10)	0.14840(12)	0.29204(3)	0.0211(4)
Cs9 (8*f*)	0.54432(8)	0.80334(10)	0.38772(3)	0.0187(4)
Rb1 (8*f*)	0.55104(9)	0.44156(10)	0.45045(3)	0.0208(4)
Rb2 (8*f*)	0.56351(9)	0.17060(10)	0.37361(3)	0.0174(4)
Rb3 (8*f*)	0.11571(9)	0.77817(11)	0.28071(3)	0.0221(4)
Rb4 (8*f*)	0.71341(9)	0.32778(10)	0.55378(3)	0.0199(4)
Rb5 (8*f*)	0.28619(9)	0.03175(10)	0.38424(3)	0.0204(4)
Rb6 (8*f*)	0.27433(9)	0.30508(10)	0.45680(3)	0.0181(4)
Rb7 (8*f*)	0.11739(9)	0.43168(10)	0.21677(3)	0.0204(4)
Rb8 (8*f*)	0.11372(10)	0.14840(12)	0.29204(3)	0.0211(4)
Rb9 (8*f*)	0.54432(8)	0.80334(10)	0.38772(3)	0.0187(4)
Tl1B (8*f*)	0.1738(14)	0.5150(20)	0.3131(5)	0.012(4)
Tl3B (8*f*)	0.3134(13)	0.2958(15)	0.3535(4)	0.013(3)
Tl5B (8*f*)	0.3128(13)	0.5510(15)	0.3796(4)	0.013(3)
Tl6B (8*f*)	0.3562(13)	0.7051(15)	0.3196(4)	0.013(3)
Tl8B (8*f*)	0.3680(20)	0.4420(30)	0.2943(7)	0.038(6)

9.3 Displacement Parameters

Table S12: Anisotropic Displacement Parameters for $Cs_{0.82}K_{0.18}Tl$. The Anisotropic displacement factor exponent takes the form: $-2\pi^2[h^2a^{*2}U_{11}+2hka^*b^*U_{12}+\ldots]$.

Atom	U_{11}	U_{22}	U_{33}	U_{23}	U_{13}	U_{12}
Tl1	0.0132(4)	0.0158(4)	0.0216(3)	-0.0014(2)	-0.0001(3)	-0.0010(2)
Tl2	0.0196(3)	0.0158(3)	0.0131(3)	0.0020(2)	0.0041(2)	-0.0016(2)
Tl3	0.0167(3)	0.0134(3)	0.0218(3)	-0.0042(2)	0.0004(2)	0.0012(2)
Tl4	0.0107(3)	0.0126(3)	0.0197(3)	-0.0018(2)	-0.0001(2)	-0.0004(2)
Tl5	0.0220(4)	0.0221(3)	0.0131(3)	0.0034(2)	0.0060(2)	-0.0003(3)
Tl6	0.0151(3)	0.0117(3)	0.0205(3)	-0.0026(2)	-0.0004(2)	0.0003(2)
Tl7	0.0133(3)	0.0143(3)	0.0225(3)	0.0018(2)	-0.0021(2)	-0.0012(2)
Tl8	0.0200(4)	0.0207(3)	0.0119(3)	0.0024(2)	0.0049(2)	-0.0016(2)
Tl9	0.0162(3)	0.0081(3)	0.0190(3)	-0.0022(2)	0.0012(2)	0.0004(2)
Cs1	0.0212(7)	0.0201(6)	0.0213(6)	0.0010(4)	0.0040(4)	-0.0011(4)
Cs2	0.0189(7)	0.0163(6)	0.0172(6)	0.0036(4)	0.0029(4)	0.0026(4)
Cs3	0.0202(7)	0.0243(7)	0.0210(6)	0.0060(4)	-0.0011(4)	-0.0001(4)
Cs4	0.0184(7)	0.0164(6)	0.0244(6)	-0.0013(4)	-0.0001(4)	-0.0041(4)
Cs5	0.0205(7)	0.0228(6)	0.0182(6)	0.0003(4)	0.0034(4)	0.0011(4)
Cs6	0.0192(7)	0.0171(6)	0.0177(6)	0.0044(4)	0.0008(4)	0.0019(4)
Cs7	0.0207(7)	0.0214(6)	0.0198(6)	0.0022(4)	0.0051(4)	0.0002(4)
Cs8	0.0224(8)	0.0214(7)	0.0197(7)	-0.0038(5)	0.0039(5)	-0.00060(5)
Cs9	0.0192(7)	0.0156(6)	0.0210(6)	-0.0037(4)	0.0010(4)	-0.0031(4)
Rb1	0.0212(7)	0.0201(6)	0.0213(6)	0.0010(4)	0.0040(4)	-0.0011(4)
Rb2	0.0189(7)	0.0163(6)	0.0172(6)	0.0036(4)	0.0029(4)	0.0026(4)
Rb3	0.0202(7)	0.0243(7)	0.0210(6)	0.0060(4)	-0.0011(4)	-0.0001(4)
Rb4	0.0184(7)	0.0164(6)	0.0244(6)	-0.0013(4)	-0.0001(4)	-0.0041(4)
Rb5	0.0205(7)	0.0228(6)	0.0182(6)	0.0003(4)	0.0034(4)	0.0011(4)
Rb6	0.0192(7)	0.0171(6)	0.0177(6)	0.0044(4)	0.0008(4)	0.0019(4)
Rb7	0.0207(7)	0.0214(6)	0.0198(6)	0.0022(4)	0.0051(4)	0.0002(4)
Rb8	0.0224(8)	0.0214(7)	0.0197(7)	-0.0038(5)	0.0039(5)	-0.0060(5)
Rb9	0.0192(7)	0.0156(6)	0.0210(6)	-0.0037(4)	0.0010(4)	-0.0031(4)

9.4 Distances

Table S13: Atomic distances in the compound $Cs_{0.82}Rb_{0.42}Tl$ (d(A-Tl)<4.75 Å, d(Tl-Tl)<3.6 Å, d(A-A)<5.45 Å).

Tl1 (8*f*)		Tl2 (8*f*)		Tl3 (8*f*)	
Tl3	3.435(1)	Tl4	3.049(1)<br>3.0596(9)	Tl5	3.0696(9)
Tl5	3.025(1)	Tl9	3.0453(9)<br>3.0672(9)	Tl7	3.3775(9)
Tl6	3.428(1)	Cs1/Rb1	4.3463(13)	Tl8	3.036(10)
Tl8	3.041(1)	Cs2/Rb2	3.7739(13)	Tl1B	3.78(2)
Tl1B	0.60(2)	Cs4/Rb4	4.1117(14)	Tl3B	1.755(17)
Tl3B	3.12(2)	Cs5/Rb5	4.2786(14)	Tl8B	2.10(3)
Tl5B	3.37(2)	Cs6/Rb6	4.5217(14)<br>4.5956(14)	Cs2/Rb	3.7697(14)
Tl8B	3.06(3)	Cs9/Rb9	4.0480(13)	Cs3/Rb3	3.9882(14)<br>4.1352(15)
Cs2/Rb2	3.8237(15)			Cs5/Rb5	4.0921(14)
Cs3/Rb3	3.8215(15)			Cs7/Rb7	4.0527(13)
Cs7/Rb7	3.940(2)<br>4.185(1)			Cs8/Rb8	3.7907(16)
Cs8/Rb8	3.6724(16)				
Cs9/Rb9	4.0968(15)				
**Tl4 (8*f*)**		**Tl5 (8*f*)**		**Tl6 (8*f*)**	
Tl9	3.3753(9)<br>3.4088(9)	Tl6	3.380(1)(4)	Tl7	3.380(1)
Cs1/Rb1	4.1988(14)	Tl7	3.0243(9)	Tl8	3.0243(9)
Cs4/Rb4	3.8092(13)<br>3.9973(14)	Tl1B	3.14(2)(6)	Tl1B	2.99(2)
Cs5/Rb5	4.0010(13)	Tl3B	1.639(17)	Tl5B	1.925(17)
Cs6/Rb6	3.8083(13)<br>3.9082(14)	Tl5B	1.475(17)	Tl6B	1.421(17)
		Tl8B	3.48(3)	Tl8B	3.61(3)
		Cs1/Rb1	4.4582(14)	Cs2/Rb2	3.8517(14)
		Cs2/Rb2	4.4623(14)<br>4.6559(14)	Cs3/Rb3	3.9035(14)
		Cs4/Rb4	4.0990(14)	Cs4/Rb4	4.0425(15)
		Cs5/Rb5	4.374(1)	Cs5/Rb5	4.2105(13)
		Cs6/Rb6	3.686(1)	Cs7/Rb7	4.0681(14)
		Cs9/Rb9	4.078(1)	Cs9/Rb9	3.7826(14)
**Tl7 (8*f*)**		**Tl8 (8*f*)**		**Tl9 (8*f*)**	
Tl8	3.1077(10)	Tl1B	2.88(2)	Cs1/Rb1	4.0194(13)<br>4.2232(13)
Tl3B	3.357(18)	Tl3B	3.743(17)	Cs4/Rb4	3.8048(14)
Tl5B	3.004(18)	Tl5B	3.748(17)	Cs6/Rb6	3.7991(14)<br>3.9197(14)
Tl6B	3.358(17)	Tl6B	2.291(17)	Cs9/Rb9	3.9415(13)
Tl8B	2.92(3)	Tl8B	0.99(3)		
Cs1/Rb1	3.9586(14)	Cs3/Rb3	4.0988(15)		

			4.4332(14)<br>4.7239(15)		
Cs2/Rb2	3.7259(13)	Cs7/Rb7	4.4610(14)<br>4.5296(13)		
Cs3/Rb3	4.3129(15)	Cs8/Rb8	3.7090(14)<br>3.9373(16)		
Cs5/Rb5	4.2864(14)				
Cs8/Rb8	3.6935(15)				
Cs9/Rb9	3.8391(13)				
Cs1/Rb1 (8*f*)		Cs2/Rb2 (8*f*)		Cs3/Rb3 (8*f*)	
Cs1/Rb1	4.640(2)	Tl1B	3.54(2)	Tl1B	3.30(2)
Cs2/Rb2	4.3754(17)	Tl3B	3.869(18)	Tl6B	3.740(18)
Cs4/Rb4	4.5536(17)<br>4.5878(17)<br>4.7626(17)	Tl5B	3.816(19)	Tl8B	3.60(3)<br>4.11(3)
Cs5/Rb5	4.672(2)	Cs3/Rb3	4.1240(16)	Cs3/Rb3	3.944(2)
Cs6/Rb6	4.3042(18)<br>5.1531(16)<br>5.1693(17)	Cs4/Rb4	4.1241(17)	Cs5/Rb5	5.441(2)
Cs9/Rb9	4.778(2)	Cs5/Rb5	4.3537(18)<br>5.1428(17)	Cs7/Rb7	4.203(2)<br>4.668(2)<br>5.133(2)
		Cs7/Rb7	5.0387(16)	Cs8/Rb8	4.161(2)<br>5.355(2)
		Cs9/Rb9	4.1552(16)		
Cs4/Rb4 (8*f*)		Cs5/Rb5 (8*f*)		Cs6/Rb6 (8*f*)	
Tl5B	3.097(17)	Tl3B	3.24(2)	Tl3B	4.31(2)
Cs4/Rb4	4.9411(18)	Cs6/Rb6	4.269(2)	Tl5B	4.260(17)
Cs5/Rb5	4.7448(16)	Cs7/Rb7	4.629(2)	Cs6/Rb6	3.870(2)
Cs6/Rb6	4.1287(16)	Cs8/Rb8	4.467(2)	Cs9/Rb9	4.107(2)
Cs9/Rb9	4.8584(18)	Cs9/Rb9	4.500(2)<br>4.635(2)		
Cs7/Rb7 (8*f*)		Cs8/Rb8 (8*f*)		Cs9/Rb9 (8*f*)	
Tl1B	4.034(19)<br>4.34(2)	Tl1B	4.25(2)	Tl1B	4.42(2)
Tl6B	2.979(17)	Tl3B	3.960(18)	Tl1B (8*f*)	
Tl8B	4.53(2)	Tl6B	4.045(18)<br>4.66(2)	Tl3B	3.46(3)
Cs7/Rb7	4.564(2)	Tl8B	4.24(3)	Tl5B	3.21(3)
Cs8/Rb8	4.413(2)<br>4.579(2)<br>4.617(2)	Cs8/Rb	4.4751(18)	Tl6B	3.37(3)
Cs9/Rb9	4.8439(17)	Cs9/Rb9	4.4832(18)	Tl8B	3.09(4)
Tl3B (8*f*)		Tl5B (8*f*)		Tl6B (8*f*)	
Tl5B	3.04(2)	Tl6B	3.11(2)	Tl8B	3.13(3)
Tl8B	3.09(3)	Tl8B	3.85(3)		

10 Effect of Disorder on the Electronic Structure of $K_{0.542}Rb_{0.458}Tl$

In the following Table S14, the atomic coordinates for the ordered system are given. The unit cell parameters are *a*=16.222128 Å, *b*=16.125015 Å, *c*=8.246807 Å, α=89.986755 °, β=90.000000 °, and γ=90.00000 °. The space group was *P*1.

Table S14: Atomic coordinates of calculation on the ordered system of $K_{0.542}Rb_{0.458}Tl$.

Atom Site Label	s.o.f.	*x*	*y*	*z*
K1	1.0	0.248312	0.229889	0.248286
K2	1.0	0.750528	0.767795	0.749416
K3	1.0	0.752163	0.268338	0.750099
K4	1.0	0.250215	0.729231	0.250415
K5	1.0	0.752163	0.731662	0.249901
K6	1.0	0.250215	0.270769	0.749585
K7	1.0	0.248312	0.770111	0.751714
K8	1.0	0.750528	0.232205	0.250584
K9	1.0	0.818054	0.000000	0.000000
K10	1.0	0.182607	0.000000	0.000000
K11	1.0	0.183149	0.500000	0.500000
K12	1.0	0.817868	0.500000	0.500000
K13	1.0	0.316452	0.500000	0.000000
Rb1	1.0	0.681661	0.500000	0.000000
Rb2	1.0	0.683540	0.000000	0.500000
Rb3	1.0	0.316962	0.000000	0.500000
Rb4	1.0	0.001294	0.195835	0.935610
Rb5	1.0	0.001294	0.804165	0.064390
Rb6	1.0	0.001044	0.304890	0.435462
Rb7	1.0	0.001044	0.695110	0.564538
Rb8	1.0	0.498925	0.697146	0.932332
Rb9	1.0	0.498925	0.302854	0.067668
Rb10	1.0	0.499859	0.802680	0.432013
Rb11	1.0	0.499859	0.197320	0.567987
Tl1	1.0	0.115209	0.394018	0.055611
Tl2	1.0	0.889062	0.607084	0.943532
Tl3	1.0	0.888657	0.107200	0.554699
Tl4	1.0	0.112651	0.892683	0.444324
Tl5	1.0	0.888657	0.892800	0.445301
Tl6	1.0	0.112651	0.107317	0.555676
Tl7	1.0	0.115209	0.605982	0.944389
Tl8	1.0	0.889062	0.392916	0.056468
Tl9	1.0	0.614052	0.893671	0.055255
Tl10	1.0	0.385659	0.105899	0.944228
Tl11	1.0	0.383794	0.607489	0.560157
Tl12	1.0	0.611999	0.395413	0.446693
Tl13	1.0	0.383794	0.392511	0.439843
Tl14	1.0	0.611999	0.604587	0.553307
Tl15	1.0	0.614052	0.106329	0.944745
Tl16	1.0	0.385659	0.894101	0.055772
Tl17	1.0	0.001001	0.531729	0.217199

Tl18	1.0	0.001001	0.468271	0.782801
Tl19	1.0	0.000781	0.968711	0.717857
Tl20	1.0	0.000781	0.031289	0.282143
Tl21	1.0	0.500021	0.032021	0.214849
Tl22	1.0	0.500021	0.967979	0.785151
Tl23	1.0	0.494627	0.467469	0.715663
Tl24	1.0	0.494627	0.532531	0.284337

11 Ground State Formation Energy Calculations of $K_{0.667}Rb_{0.333}Tl$

Table S15: Formation energy values and the site occupancy factors of the three different alkali metal positions in the KTl structure type, depicted in Figure 10 in the main manuscript.

	Formation Energy /eV	*A*1	*A*2	*A*3
1	-81.36821922	Rb	K	K
2	-81.42041955	K	Rb	K
3	-81.57414500	K	K	Rb

12 Details on Phonon Calculations

Table S16: Details on Phonon Calculations.

	RbTl
Space group	*Cmce* (KTl)
Super cell	2x2x2
Symmetry for calculation	*Cmce* (No. 64)

In Table S16, the applied unit cell for calculation of the phonons are given, and their relation to the original unit cell.

References

(1) Dong, Z. C.; Corbett, J. D. Synthesis, Structure, and Bonding of the Novel Cluster Compound KTl with Isolated Tl_6^{6-} Ions. *J. Am. Chem. Soc.* **1993**, *115* (24), 11299-11303. DOI: 10.1021/ja00077a031.

(2) Dong, Z. C.; Corbett, J. D. CsTl: A new example of tetragonally compressed Tl_6^{6-} octahedra. Electronic effects and packing requirements in the diverse structures of ATl (A=Li, Na, K, Cs). *Inorg. Chem.* **1996**, *35* (8), 2301-2306. DOI: 10.1021/ic951265v.

(3) Thümmel, R.; Klemm, W. Das Verhalten der Alkalimetalle zu den Metallen der Gruppe III B. *Z. Anorg. Allg. Chem.* **1970**, *376* (1), 44-63.

(4) Kaskel, S.; Corbett, J. D. Synthesis and structure of $K_{10}Tl_7$: The first binary trielide containing naked pentagonal bipyramidal Tl_7 clusters. *Inorg. Chem.* **2000**, *39* (4), 778-782. DOI: 10.1021/ic991168s.

(5) Schwinghammer, V. F.; Gärtner, S. $[Tl_7]^{7-}$ Clusters in Mixed Alkali Metal Thallides $Cs_{7.29}K_{5.71}Tl_{13}$ and $Cs_{3.45}K_{3.55}Tl_7$. *Inorg. Chem.* **2024**, 5, Early Access. DOI: 10.1021/acs.inorgchem.3c04034.

(6) Karpov, A.; Jansen, M. $A_{10}Tl_6O_2$ (A = K, Rb) cluster compounds combining structural features of thallium cluster anions and of alkali metal sub-oxides. *Chem. Commun.* **2006**, (16), 1706-1708. DOI: 10.1039/b601802e.

(7) Saltykov, V.; Nuss, J.; Jansen, M. $Cs_{10}Tl_6SiO_4$, $Cs_{10}Tl_6GeO_4$, and $Cs_{10}Tl_6SnO_3$ - First Oxotetrelate Thallides, Double Salts Containing "Hypoelectronic" Tl_6^{6-} Clusters. *Z. Anorg. Allg. Chem.* **2011**, *637* (9), 1163-1168. DOI: 10.1002/zaac.201000358.

(8) Abrikosov, I.; Johansson, B. Applicability of the coherent-potential approximation in the theory of random alloys. *Phys. Rev. B* **1998**, *57* (22), 14164-14173.
